# From Invaginating Site to Deep Lesion: Spatial Transcriptomics Unravels Ectopic Endometrial Penetration Features in Adenomyosis

**DOI:** 10.1002/advs.202411752

**Published:** 2025-04-07

**Authors:** Boyu Li, Jia Qi, Yumeng Cao, Yijing Long, Zhe Wei, Wang‐Sheng Wang, Shuanggang Hu, Yuan Wang, Qinling Zhu, Xiao Hu, Zhe Sun, Jie Zhu, Taiyang Ye, Yejie Yao, Yiwen Meng, Xuejiao Bian, Xinyi Dong, Hengyu Guan, Yunfei Huang, Yun Sun

**Affiliations:** ^1^ Department of Reproductive Medicine Ren Ji Hospital Shanghai Jiao Tong University School of Medicine Shanghai 200135 P. R. China; ^2^ Shanghai Key Laboratory for Assisted Reproduction and Reproductive Genetics Shanghai 200135 P. R. China; ^3^ Department of Obstetrics and Gynecology Ren Ji Hospital Shanghai Jiao Tong University School of Medicine Shanghai 200127 P. R. China; ^4^ Shanghai Key Laboratory of Gynecologic Oncology Shanghai 200127 P. R. China

**Keywords:** adenomyosis, fibrosis, invagination, invasion, spatial transcriptomics

## Abstract

Adenomyosis, characterized by clinical intractability, significantly impacts female fertility and life quality due to the absence of definitive diagnostic markers and effective treatment options. The invagination theory is a primary hypothesis for adenomyosis, but the underlying molecular mechanisms remain unclear. In this study, a spatial transcriptional landscape of adenomyosis with an evident invagination structure is mapped from the endometrial invaginating site to ectopic lesions utilizing spatial transcriptomics and single‐cell RNA sequencing. In addition, the authors employ bulk RNA sequencing deconvolution to assess the significance of core spatial ecotypes, use histological techniques to target specific cell types, and conduct in vitro experiments for validation. At the invagination site, SFRP5^+^ epithelial cells promote endometrial proliferation and angiogenesis through secretion of IHH. During the invading process, ESR1^+^ smooth muscle cells (SMCs) facilitate invasion by creating migratory tracts via collagen degradation. Within deep lesions, CNN1^+^ stromal fibroblasts induce fibrosis by undergoing a fibroblast‐to‐myofibroblast transition (FMT) in response to pathologic profibrogenic signals in the microenvironment of lesions. This work offers an in‐depth understanding of the molecular mechanisms underlying the pathological processes of adenomyosis with invagination. Furthermore, this work introduces the first transcriptomics web source of adenomyosis, which is expected to be a valuable resource for subsequent research.

## Graphical abstract

This work utilizes spatial transcriptomics to map the transcriptional landscape of adenomyosis with obvious invagination structure from the invaginating site to deep lesions. By integrating with single‐cell RNA sequencing, this study elucidates the molecular features of invagination, invasion, and fibrosis, and finds the major participants and vital cell–cell communication responsible for these processes.

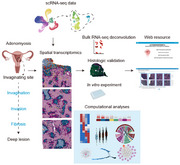

## 1 Introduction

Adenomyosis is defined by ectopic endometrial glands and stroma within the myometrium.^[^ ^1^ ^]^ The regular ectopic endometrial hemorrhage causes fibrogenesis around the lesions and an indistinct boundary with the hypertrophic and hyperplastic myometrium.^[^ ^2^ ^]^ With typical symptoms including dysmenorrhea, menorrhagia, chronic pelvic pain, and infertility, adenomyosis impairs women's fertility and general quality of life.^[^ ^3^ ^]^ Nevertheless, adenomyosis persists as an enigmatic disease, owing to its understudied etiology and pathogenesis. Due to the absence of specific laboratory tests and effective medical therapies, there is an urgent need to enhance our understanding of the pathophysiology of adenomyosis.^[^ ^4^ ^]^

The invagination theory proposes that adenomyosis lesions result from the invagination and invasion of the endometrial basalis into the myometrium, which is one of the most widely accepted explanations for the initiation of adenomyosis.^[^ ^5^ ^]^ Imbalance at the endometrial–myometrial interface is considered as the primary event in the etiology of adenomyosis.^[^ ^5^, ^6^ ^]^ The higher occurrence of adenomyosis in parous women and those who have had one or more spontaneous abortions supports the invagination hypothesis.^[^ ^7^ ^]^ Moreover, targeted deep sequencing of adenomyosis revealed the presence of recurring *KRAS* mutations in adenomyosis lesions and adjacent basalis endometrial glands, supporting the basalis endometrial origination of adenomyosis lesions.^[^ ^8^, ^9^ ^]^ Targeted deep sequencing of adenomyosis revealed that somatic mutations in adenomyosis resided in epithelial cells,^[^ ^8^, ^9^ ^]^ suggesting that epithelial cells may play an important role in the process of invagination. Previous studies have elucidated the role of epithelial cells in the progression of adenomyosis lesions. Epithelial cells propel the invasion and migration of lesions through epithelial‐to‐mesenchymal transition (EMT),^[^ ^6^ ^]^ enhance local estradiol levels by reducing estradiol inactivation process,^[^ ^7^ ^]^ facilitate lesion development via the WNT pathway,^[^ ^10^, ^11^ ^]^ and promote vasculogenic mimicry in lesions through epithelial‐endothelial transition.^[^ ^12^ ^]^ But the alteration in gene expression of epithelial cells, and the signaling network with other cells during the invagination process remain unknown.^[^ ^7^ ^]^ The progression of adenomyosis lesions is delineated across three pivotal stages, distinguished by their spatial characteristics: invagination at the endometrial–myometrial interface, inside‐to‐outside invasion, and lesion fibrosis.^[^ ^2^, ^7^, ^13^ ^]^ However, the initiating factors of endometrial invagination, the driving factors of lesion invasion, and the propelling factors of fibrosis within deep lesions remain unclear.

Recently, sequencing tools have provided a more comprehensive understanding of the molecular pathogenesis of adenomyosis. Microarray^[^ ^14^, ^15^ ^]^ and RNA sequencing^[^ ^16^, ^17^, ^18^, ^19^, ^20^ ^]^ of adenomyosis at various times of the menstrual cycle demonstrated overall transcriptional changes of adenomyosis. Single‐cell transcriptomics enables us to monitor physiological and pathological processes in adenomyosis at a cellular level and find lesion‐specific cells.^[^ ^10^, ^12^ ^]^ By integrating single‐cell RNA sequencing and spatial transcriptomics, Che et al. revealed lesion‐specific stem cell‐like and invasive cell subpopulations and their spatial locations.^[^ ^11^ ^]^ Their results also supported the metaplasia theory, another major theory for the etiology of adenomyosis.^[^ ^5^ ^]^ Spatial transcriptomics offers distinct advantages for investigating diseases characterized by spatial heterogeneity.^[^ ^21^, ^22^ ^]^ Given that invagination, invasion, and fibrosis are processes with distinct histological features and significant spatial architecture, the integration of spatial transcriptomics with single‐cell RNA sequencing is a potent approach to explore the molecular underpinnings of these processes, thereby enhancing our understanding of adenomyosis with invagination.

This study used spatial transcriptomics to investigate the transcriptional alterations from invaginating site to deep lesions of adenomyosis, depicting a spatial molecular landscape of invagination, invasion, and fibrosis. By integrating previously reported single‐cell RNA sequencing data,^[^ ^12^, ^23^, ^24^ ^]^ we found the adenomyosis‐related spatial ecotypes (groups of spatially colocalized and phenotypically coherent cell states^[^ ^22^ ^]^), and explored the spatial transcriptional alterations and divergent trajectory of major spatial ecotypes in endometrium (predominantly composed of epithelial cells or stromal fibroblasts) and myometrium (predominantly composed of smooth muscle cells, SMCs). This study provides a new perspective for understanding the mechanisms underlying invagination‐related adenomyosis, prompting further investigation of potential noninvasive diagnostic and therapeutic targets. Furthermore, we developed a publicly accessible digital resource for this field (https://sylabamcellatlas.sjtu.edu.cn/).

## 2 Results

### 2.1 Spatial Transcription Mapping of Human Adenomyosis

**Figure 1:**
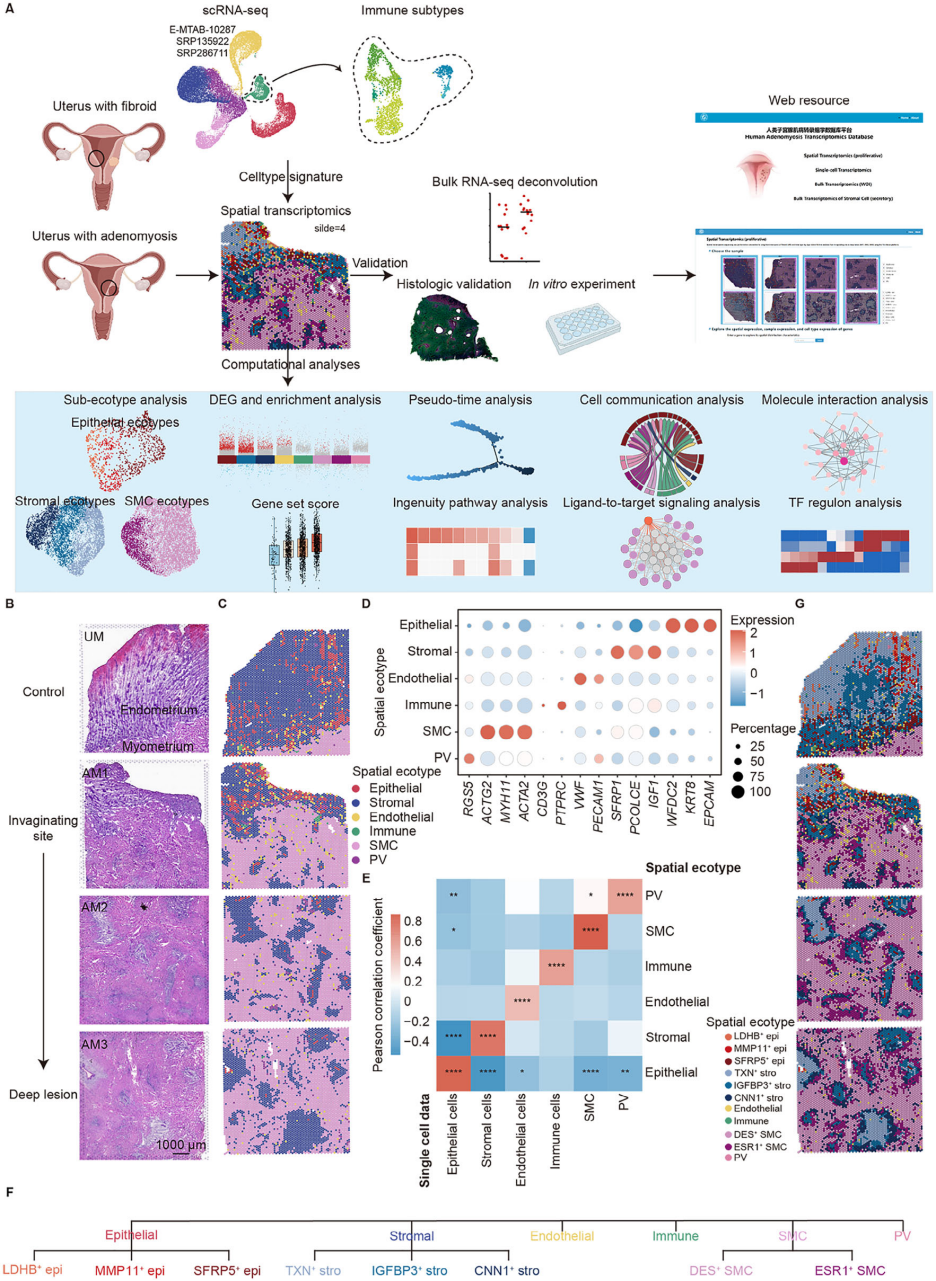
Spatial transcriptomics of human adenomyosis. A) Illustration of study workflow. Uterine tissues were collected from women with adenomyosis and fibroid. One section far away from the leiomyoma of fibroid and three sequential sections from adenomyosis with obvious invagination structure were selected for 10x Visium spatial transcriptomic sequencing. Single‐cell data from published studies on control (healthy women or women with fibroid) and women with adenomyosis were integrated for cell type deconvolution. Validation was conducted using bulk RNA‐seq deconvolution, histologic methods, and in vitro experiments. A web platform for adenomyosis transcriptomics database was developed for further investigation. The computational analysis of spatial transcriptome data is described in the blue square. TF, transcription factor. B) H&E staining of four tissue sections used for spatial transcriptomics. The junction of the endometrium and myometrium is delineated with a white dashed line. “UM” denotes a section situated at a considerable distance from the leiomyoma, whereas “AM1‐AM3” denotes three layer‐by‐layer sections from invaginating site to deep lesions. Scale bars: 1000 µm. C) Spatial ecotypes after Cell2location. The spatial transcriptomics map is colored by six spatial ecotypes. D) Dot plots showing the average expression of known markers in indicated ecotypes. E) The Pearson correlation heatmap of DEG expression between the 10x Visium spatial transcriptome data and the single‐cell data with the corresponding cell types. *p < 0.05, **p < 0.01, ****p < 0.0001. F) Tree diagram showing the hierarchy of ecotype annotation in the Visium data. The first level of the tree represents six ecotypes annotated by Cell2location. The leaves represent the further unsupervised clustering of epithelial ecotypes, stromal ecotypes, and SMC ecotypes. G) Spatial transcriptomics map.

For spatial transcriptomic sequencing and subsequent validation, we established a bank of uterine samples from hysterectomy for adenomyosis (endometrium and myometrium tissue around adenomyosis lesion, as shown in black circle) or fibroid (tissue far away from leiomyoma, as shown in black circle) under strict inclusion and exclusion criteria (**Figure** **1**A; Data S1, Supporting Information; adenomyosis, *n* = 16; fibroid, *n* = 11). For the definition of spatial ecotypes, published single‐cell RNA sequencing data of uterine tissues from the control^[^ ^23^, ^24^ ^]^ and adenomyosis patients^[^ ^12^ ^]^ was integrated by the canonical correlation analysis (CCA) (Figure S1A,B, Supporting Information), and re‐analyzed to generate cell type signatures (*n* = 48036). We obtained the gene expression profiles of six main cellular categories: epithelial cells (*n* = 8385), stromal fibroblasts (*n* = 13254), endothelial cells (*n* = 9193), immune cells (*n* = 3916), SMCs (*n* = 5291), and pericytes (PV, *n* = 7997) as reference input data of Cell2location^[^ ^25^ ^]^ for spot deconvolution (Figure S1C–F and Data S2, Supporting Information).

To investigate the dynamic change of adenomyosis from the invaginating site to deep lesions, we selected three layer‐by‐layer 6.5 mm × 6.5 mm sections (slice AM1‐3) from one adenomyosis patient and one section (slice UM) far away from leiomyoma of a fibroid patient as control (Figure 1B). The adenomyosis section was featured with typical morphological endometrial invagination (Figure 1B). We chose the 10x Genomics Visium platform (capture RNA from spots with diameters of 55 µm) for spatial transcriptomic sequencing. A total of 18478 spots were detected. The mean sequencing depth was 73609 reads per spot (range = 65627–78577), with an average UMI count of 8905 (range = 7497–10591) and a median gene number of 3296 (range = 2828–3498) per spots (Figure S2A and Data S3, Supporting Information). Cell2location was used to quantify individual cell type abundance across locations, and the spatial spots were annotated using the cell type with the largest proportion (Data S4, Supporting Information). We defined the spots with the same largest‐proportion cell type as a group of spatial ecotypes (Figure 1C). To check the association of the major cell type with the spatial ecotype's phenotypic states, we explored the expression levels of cell type markers in respective annotated spatial ecotypes as follows: *EPCAM*, *KRT8*, and *WFDC2* for epithelial ecotypes (*n* = 991); *IGF1*, *PCOLCE*, and *SFRP1* for stromal ecotypes (*n* = 7101); *PECAM1*, *VWF* for endothelial ecotypes (*n* = 578); *PTPRC*, *CD3G* for immune ecotypes (*n* = 70); *ACTA2*, *MYH11*, and *ACTG2* for SMC ecotypes (*n* = 9356); *RGS5* for PV ecotypes (*n* = 382). The spatial expression of these cell type markers in the uterus was validated in the Human Protein Atlas database (Figure S2B, Supporting Information). Spatial ecotypes showed specific high expression levels of the markers of their corresponding cell types (Figure 1D). Pearson correlation analysis between the spatial ecotypes and the single cells with the same cell type annotation exhibited strong correlation (Pearson correlation coefficient = 0.6905(±0.1665),*p*‐value < 0.0001), indicating the largest‐proportion cell type played a major role in respective spatial ecotypes (Figure 1E). The spatial expression of each cell type marker was consistent with the distribution of corresponding spatial ecotypes (Figure 1C; Figure S2C, Supporting Information), which further verified the cell type annotation.

To take a macroscopic view of these spatial ecotypes in adenomyosis, we first explored the proportions of these six spatial ecotypes across slices and the differential expression genes (DEGs) between adenomyosis and control in each ecotype. Epithelial ecotypes and stromal ecotypes were the main ecotypes in the endometrium, and myometrium was predominantly composed of SMC ecotypes (Figure S3A, Supporting Information). As for gene expression, the most significant differences were also observed in the epithelial ecotypes, stromal ecotypes, and SMC ecotypes (Figure S3B, Supporting Information). The ectopic endometrium demonstrates a dynamic and progressive nature, with several cell types undergoing significant changes throughout the natural history of adenomyosis, displaying distinct characteristics within their respective microenvironments.^[^ ^26^ ^]^ Therefore, to further decipher the imbalance of endometrium and myometrium and the molecular characteristics of microenvironments, we re‐clustered epithelial ecotypes, stromal ecotypes, and SMC ecotypes, and annotated the sub‐ecotypes with their specific genes (Figure S3C–H and Data S5, Supporting Information). Sub‐ecotypes of the three spatial ecotypes exhibited distinct features in spatial distribution (Figure 1F,G), demonstrating that the changes in gene expression led to unique spatial microenvironments.

### 2.2 SFRP5^+^ Epithelial Cells Participate in Invagination by Expressing IHH

**Figure 2:**
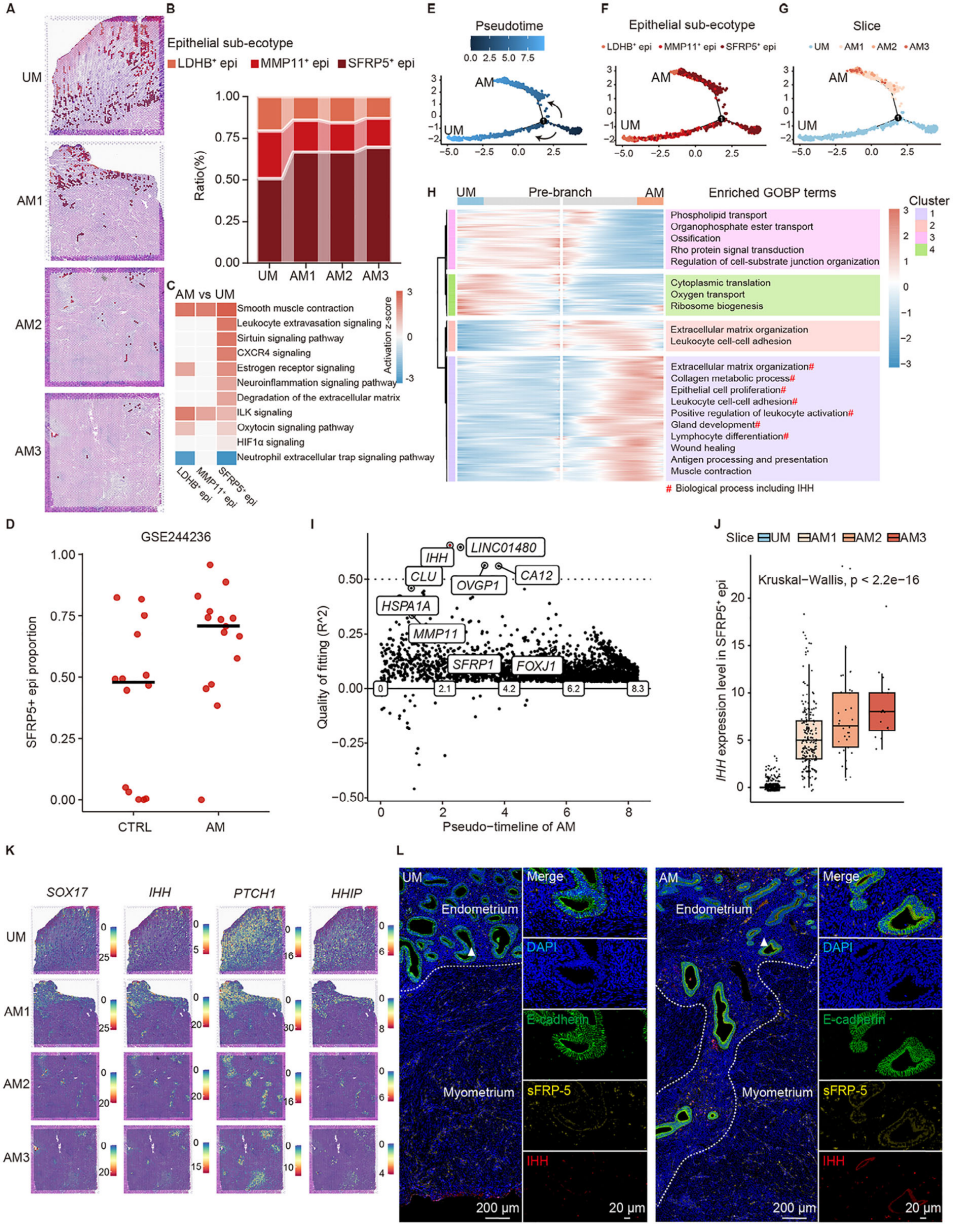
Identification of sub‐ecotypes and trajectory inference within the epithelial ecotypes. A) Location of three epithelial sub‐ecotypes. The color of epithelial‐ecotype spots refers to (B). B) Percentage of these three epithelial sub‐ecotypes in different slices. C) Heatmap showing IPA activation score of signaling pathway related to adenomyosis in each epithelial sub‐ecotype. DEGs was obtained from the DESeq2 differential expression analysis between groups (AM1‐3 as group AM; UM as group UM) in corresponding epithelial sub‐ecotypes. D) Estimated SFRP5^+^ epithelial ecotype proportion in bulk RNA‐seq data. E‐H) Pseudotime trajectory of epithelial ecotypes constructed by Monocle. Trajectory colored by pseudotime (E), epithelial sub‐ecotypes (F) and slices (G), and GOBP terms significantly enriched in each gene cluster with different pseudo‐temporal expression patterns (H) are shown. The statistical analysis was performed by Fisher's test. GOBP, biological processes terms in gene ontology resource. I) Switching genes along the pseudo‐timeline of AM in (E‐G). The genes that act as on/off switches were estimated by GeneSwitches. J) Box plot showing *IHH* expression level in SFRP5^+^ epithelial ecotypes in different slices. Data are presented as the quartiles. The Kruskal‐Wallis rank sum test was used to obtain p‐values. K) IHH‐related gene expression visualization on top of tissue histology. *SOX17*, *PTCH1*, and *HHIP* are respectively transcription factors, receptors, and target genes of *IHH*. L) Representative mIHC staining images of adenomyosis (AM) and control (UM) showing the expression of ECAD (epithelial marker), SFRP5, and IHH. The junction of the endometrium and myometrium is delineated with a white dashed line. White triangles indicate the endometrium‐myometrium junction of control (left) and the invaginating site of adenomyosis (right) shown at higher magnification. Scale bars: higher magnification, 20 µm; other, 200 µm.

Previous research suggested that epithelial cells played a key role in the development of adenomyosis.^[^ ^7^, ^8^ ^]^ Our spatial map revealed three sub‐ecotypes inside the epithelial ecotypes and we performed differential expression and enrichment analysis between these sub‐ecotypes (**Figure** **2**A; Figure S4A and Data S6andS7, Supporting Information): LDHB^+^ epithelial ecotypes (*n* = 181) were found in the superficial epithelium, with a lower proportion in adenomyosis (Figure 2B; Figure S4B, Supporting Information); MMP11^+^ epithelial ecotypes (*n* = 246) were found in the middle and were associated with extracellular matrix (ECM) organization (Figure S4B,C; Data S6 and S7, Supporting Information); SFRP5^+^ epithelial ecotypes (*n* = 564) were found located closer to the myometrium (Figure S4B, Supporting Information), with a higher prevalence in adenomyosis and were involved in biological processes such as immune regulation and muscle contraction (Figure 2B; Figure S4C and Data S6 and S7, Supporting Information). To assess the roles of these epithelial sub‐ecotypes in the pathogenesis of adenomyosis, we performed differential expression analysis between the adenomyosis group and the control in these three epithelial sub‐ecotypes, and estimated the activation score (the predicted activity of the pathway based on z‐score algorithm) of ingenuity canonical pathways related to adenomyosis^[^ ^6^, ^13^, ^27^, ^28^, ^29^, ^30^ ^]^ using ingenuity pathway analysis (IPA) (Data S8, Supporting Information). Adenomyosis‐related pathways, such as leukocyte extravasation signaling, were mainly activated in SFRP5^+^ epithelial ecotypes (Figure 2C). To further investigate the correlation between the SFRP5^+^ epithelial ecotypes and adenomyosis, we employed MuSiC2^[^ ^31^ ^]^ to estimate SFRP5^+^ epithelial ecotype proportions in bulk RNA‐seq data of adenomyosis epithelial organoids^[^ ^20^ ^]^ and found a higher proportion of SFRP5^+^ epithelial ecotypes within AM group (Figure 2D). These results indicated the involvement of basal epithelium in the pathogenesis of adenomyosis and supported the invagination hypothesis, which highlighted the basalis endometrial origin of adenomyosis.

The processes of endometrial invagination and invasion were inter‐connected. To investigate the dynamic changes of epithelial ecotypes, we constructed pseudo‐temporal ordering by Monocle.^[^ ^32^, ^33^, ^34^ ^]^ The pseudo‐timelines were split into two branches, representing different groupings (Figure 2E). In the control group, SFRP5^+^ epithelial ecotypes progressed through MMP11^+^ epithelial ecotypes to LDHB^+^ epithelial ecotypes (timeline UM of Figure 2F), which was consistent with the consensus that the functional layer of the endometrium was produced by the basal layer during the menstrual cycle. In the adenomyosis group, epithelial ecotypes in slice AM1 were ordered earlier on the pseudotime line while epithelial ecotypes in slice AM3 were ordered later (timeline AM of Figure 2G), which was consistent with the progressive deepening of the adenomyosis lesions. According to branched expression analysis modeling, branch‐dependent genes involved in immune regulation, ECM organization, cell proliferation, and muscle contraction were enriched along the AM timeline (Figure 2H; Data S9, Supporting Information). Further GeneSwitches analysis, which used pseudo‐time trajectory to identify genes that operated as on/off switches and the order in which these switches occurred, showed that *IHH* was the best‐fitted gene on timeline AM (McFadden's Pseudo R^2 = 0.6555, Figure 2I). The *IHH* expression pattern in epithelial subpopulations shifted during adenomyosis. In control (group UM), *IHH* expression level decreased from LDHB^+^ epithelial ecotypes through MMP11^+^ epithelial ecotypes to SFRP5^+^ epithelial ecotypes (left of Figure S4D, Supporting Information). While in adenomyosis (group AM), *IHH* expression level abnormally increased in SFRP5^+^ epithelial ecotypes (right of Figure S4D, Supporting Information). SFRP5^+^ epithelial ecotypes showed increasing levels of *IHH* as the lesion deepened (Figure 2J). *IHH*, as well as its transcription factor, receptor, and target gene (*SOX17*, *PTCH1*, and *HHIP*, respectively), exhibiting spatial expression characteristics, were highly expressed in the invaginating site of adenomyosis (Figure 2K). Multiplex Immunohistochemistry (mIHC) labeling E‐cadherin (protein of *ECAD*, an epithelial marker), sFRP‐5 (protein of *SFRP5*), and IHH confirmed the distribution of SFRP5^+^ECAD^+^ cells in the basal layer of endometrium, which is consistent with the spatial transcriptomics data (Figure S4E, Supporting Information), and the higher expression level of IHH at the invaginating site of adenomyosis (Figure 2L), suggesting that SFRP5^+^ epithelial cells are the key players in SFRP5^+^ epithelial ecotypes. Besides, in adenomyosis samples, portions with obvious invaginating structure had a greater IHH level than parts lacking this feature (Figure S4F, Supporting Information), indicating the invagination‐related expression of IHH. Along the trajectory from AM1 to AM3, *IHH* was implicated in some important biological processes, such as ECM organization, immune regulation, and epithelial proliferation (Figure 2H), indicating its potential function.

To summarize, SFRP5^+^ epithelial cells were a group of pro‐adenomyosis epithelial cells located in the basal layer of the endometrium, exhibiting increased proportion and activation of adenomyosis‐related pathways compared to that in the control group. These findings align with the invagination theory that the lesion originates in the basalis endometrium. Abnormally high expression of IHH was spotted in the SFRP5^+^ epithelial cells of adenomyosis patients. *IHH* increased along the pseudo‐temporal trajectory of epithelial cells in adenomyosis, engaging in key processes in the adenomyosis pathogenesis such as immune regulation and ECM organization.

### 2.3 SFRP5^+^ Epithelial Cells in the Invaginating Microenvironment Stimulate Endometrial Proliferation and Angiogenesis by Secreting IHH

**Figure 3:**
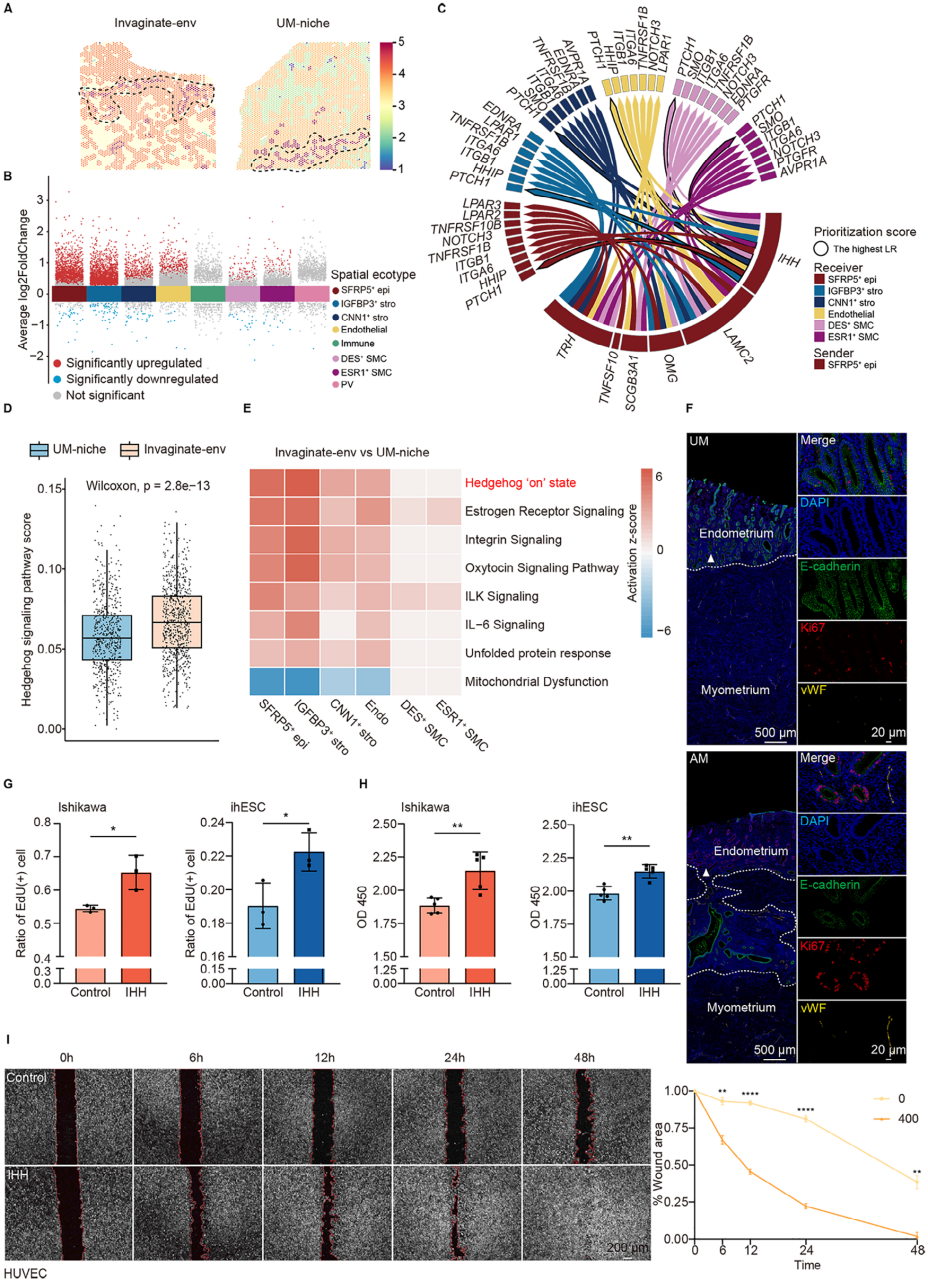
Transcriptional signature of spatial ecotypes in invaginating microenvironment. A) Cell–cell interaction activity score of spots in slice AM1 and UM by stLearn. The microenvironment region is circled by black dashed lines. Invaginate‐env and UM‐niche indicate the junction area between the endometrium and myometrium of adenomyosis and the control, respectively. B) Volcano plot showing DEGs between invaginate‐env and UM‐niche in each spatial ecotype. The statistical analysis was performed by the Wilcoxon test. C) Circos plot showing active ligand‐target signaling from SFRP5^+^ epithelial ecotypes in invaginating microenvironment according to NicheNet. The arrow points from the sender to the receiver. Black line circles ligand‐receptor pairs with the highest prioritization score in each receiver. D) Box plot showing the expression of gene set in Hedgehog signaling pathway between invaginating microenvironment and the control niche by AUCell. Data are presented as the quartiles. The Wilcoxon test was used to obtain p‐values. E) Heatmap showing activation score of canonical pathways of IPA database in each spatial ecotype in invaginating microenvironment, compared to the control niche. F) Representative mIHC staining images of adenomyosis (AM) and control (UM) showing the expression of ECAD (epithelial marker), KI67 (proliferation marker), and VWF (vascular endothelial marker). The junction of the endometrium and myometrium is delineated with a white dashed line. White triangles indicate the endometrium‐myometrium junction of control (upper) and the invaginating site of adenomyosis (bottom) shown at higher magnification. Scale bars: higher magnification, 20 µm; other, 500 µm. G‐H) EdU (G) and CCK‐8 (H) analysis of Ishikawa and ihESC after treatment with recombinant IHH for 24 h. *n* = 3 in (G); *n* = 5 in (H). Data are presented as the mean ± SD. An unpaired two‐tailed Student's t‐test was used to obtain p‐values, *p < 0.05, **p < 0.01. I) Wound healing assay of HUVEC after treatment with recombinant IHH. Scale bars: 200 µm. *n* = 3. Data are presented as the mean ± SD. The two‐way ANOVA followed by Bonferroni's multiple comparisons test was used to obtain p‐values, **p < 0.01, ****p < 0.0001.

Active cellular interactions are commonly observed at the junction site of invasive diseases.^[^ ^35^ ^]^ stLearn^[^ ^36^ ^]^ revealed high ligand–receptor interaction activity at the endometrium‐myometrium junction site (**Figure** **3**A). We labeled the junction region in adenomyosis with endometrial invagination as invaginating microenvironment (invaginate‐env; Figure 3A; Figure S5A, Supporting Information), and the control counterpart as control niches (UM‐niche; Figure 3A; Figure S5B, Supporting Information). There were eight spatial ecotypes distributed in these two microenvironments: SFRP5^+^ epithelial ecotypes, IGFBP3^+^ stromal ecotypes, CNN1^+^ stromal ecotypes, endothelial ecotypes, immune ecotypes, DES^+^ SMC ecotypes, ESR1^+^ SMC ecotypes, and PV ecotypes (Figure S5A,B, Supporting Information). Differential expression analysis was performed on these eight spatial ecotypes between these two microenvironments to investigate the transcriptional signature changes (Data S10–S15, Supporting Information). SFRP5^+^ epithelial ecotypes, stromal ecotypes, and endothelial ecotypes exhibited the most drastic transcriptional changes (Figure 3B). To identify the regulatory drivers of these changes, we performed NicheNet^[^ ^37^ ^]^ analysis to model intercellular communication (Data S16–S21, Supporting Information). *IHH*, the ligand secreted by SFRP5^+^ epithelial ecotypes, had the highest prioritization score (calculated by NicheNet^[^ ^37^ ^]^ according to the potential of ligands to regulate a set of affected genes in receivers) across all receivers (Figure 3C). Due to the limited number of DEGs, NicheNet was unable to analyze the prioritized ligands when immune ecotypes and PV ecotypes were inputted as receivers.

IHH, one of the three ligands of Hedgehog signaling, takes part in embryonic development and tumorigenesis.^[^ ^38^, ^39^ ^]^ Compared to the control niche, invaginating microenvironment showed significantly high expression of Hedgehog signaling pathway gene set by AUCell (Figure 3D). IPA was employed to estimate the activation score of ingenuity canonical pathway on DEGs between invaginating microenvironment and the control niche in each spatial ecotype. Hedgehog “on” state was activated in SFRP5^+^ epithelial ecotypes, stromal ecotypes, and endothelial ecotypes (Figure 3E). Hedgehog signaling involves three main parts: signal biogenesis, signal release and transfer, and signal intracellular transduction.^[^ ^40^ ^]^ In sender (SFRP5^+^ epithelial ecotypes), *IHH*, *HHAT* (participates in signal biogenesis^[^ ^38^ ^]^), *DISP1* and *SCUBE2* (important in release and transfer of Hedgehog^[^ ^38^ ^]^) were upregulated in invaginating microenvironment (Figure S5C, Supporting Information). In receiver (SFRP5^+^ epithelial ecotypes, stromal ecotypes, and endothelial ecotypes), receptor (encoded by *PTCH1*), co‐receptors (encoded by *GAS1*, *BOC*), and common target genes of Hedgehog signaling (encoded by *GLI1*, *GLI2*, *GLI3*, *PTCH2*, *HHIP*, *CCND1*, *CCND2*, *BCL2*, *HIP1*),^[^ ^38^ ^]^ were upregulated in invaginating microenvironment (Figure S5C, Supporting Information). These results indicated the activation of Hedgehog signaling in invaginating microenvironment. Molecule interaction analysis indicated that the Hedgehog pathway was linked to the key pathogenic bio‐processes in the invaginating microenvironment, including epithelial cell proliferation in SFRP5^+^ epithelial ecotypes (red), fibroblast proliferation in stromal ecotypes (blue), and positive regulation of angiogenesis in endothelial ecotypes (yellow) (Figure S5D, Supporting Information). Cell proliferation and angiogenesis are critical factors that promote basalis endometrium invagination.^[^ ^5^ ^]^ Previous studies have also suggested that the Hedgehog pathway induced proliferation of epithelial cells and stromal fibroblasts,^[^ ^41^, ^42^ ^]^ as well as angiogenesis,^[^ ^43^ ^]^ implying that IHH secreted by SFRP5^+^ epithelial ecotypes in the invaginating microenvironment may play an important role in adenomyosis pathogenesis via autocrine and paracrine mechanism.

mIHC labeling E‐cadherin, Ki67 (protein of *MKI67*, a proliferation marker), and vWF (protein of *VWF*, a vascular endothelial cell marker) showed a higher ratio of proliferating endometrial cells and an increased number of vascular endothelial cells in the invaginating microenvironment (Figure 3F). Using cell counting kit‐8 (CCK‐8), 5‐ethylnyl‐2'‐deoxyuridine (EdU), and wound healing assays, we subsequently validated that the cell proliferation of Ishikawa (human endometrial carcinoma cell line, the replacement for endometrial epithelial cells) and ihESC (immortalized human endometrial stromal cells) was enhanced after the administration of recombinant IHH (Figure 3G,H and Figure S5E, Supporting Information). The cell migration of HUVEC (human umbilical vein endothelial cells) was also enhanced with the treatment of IHH (Figure 3I).

In the invaginating microenvironment, SFRP5^+^ epithelial cells stimulated endometrium proliferation and angiogenesis through autocrine and paracrine of IHH, promoting adenomyosis development.

### 2.4 SFRP5^+^ Epithelial Cells Undergo Epithelial‐to‐Mesenchymal Transition (EMT) in the Invaginating Microenvironment

**Figure 4:**
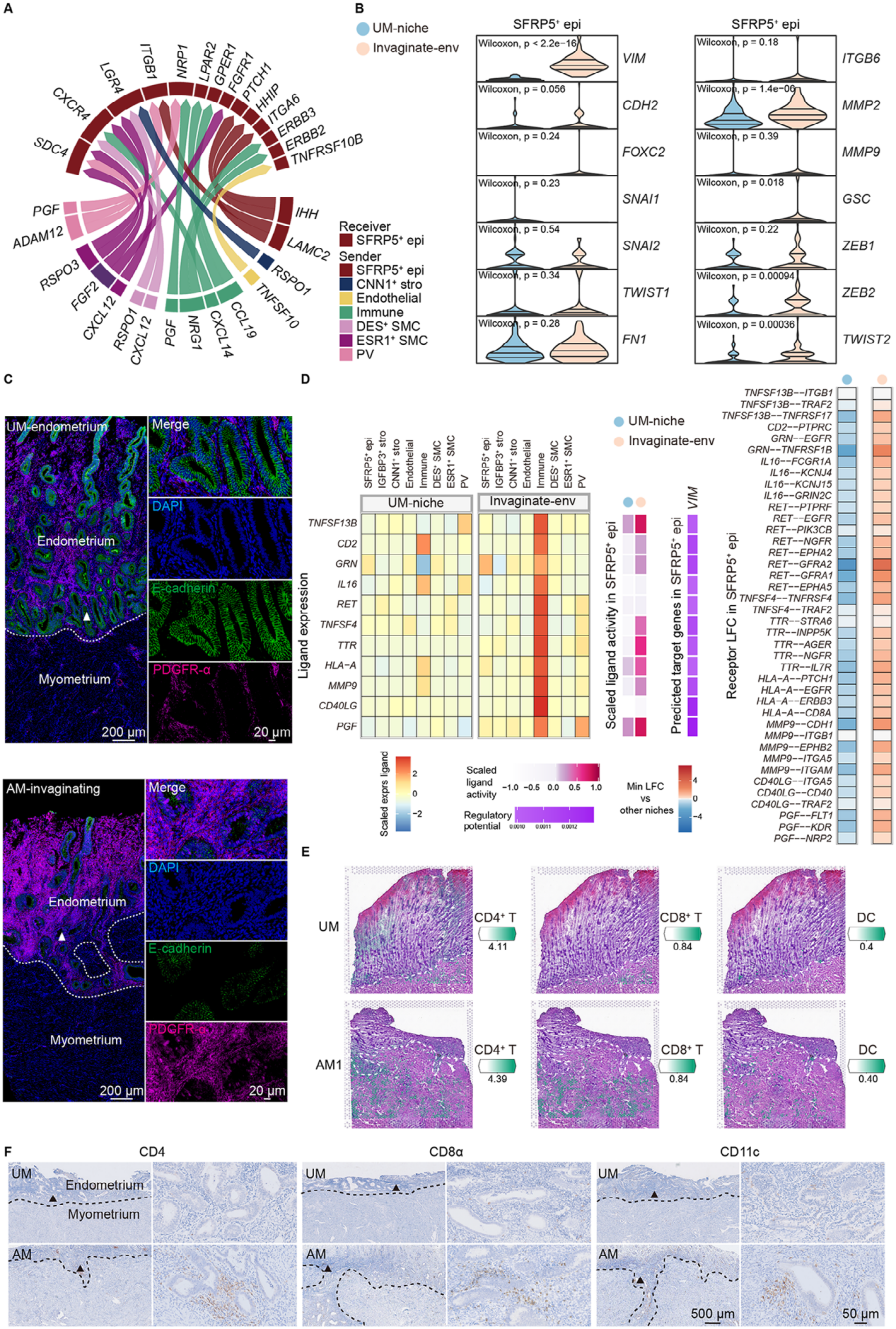
The EMT‐driving mediators in the invaginating microenvironment. A) Circos plot showing active EMT‐related ligand signals received by SFRP5^+^ epithelial ecotypes in invaginating microenvironment by NicheNet analysis. The arrow points from the sender to the receiver. B) Expression of mesenchymal marker genes in SFRP5^+^ epithelial ecotypes from invaginating microenvironment and the control niche. Data are presented as the quartiles. The Wilcoxon test was used to obtain p‐values. C) Representative mIHC staining images of adenomyosis (AM‐invaginating) and control (UM‐endometrium) showing the expression of ECAD (epithelial marker) and PDGFRA (stromal fibroblast marker). The junction of the endometrium and myometrium is delineated with a white dashed line. White triangles indicate the endometrium‐myometrium junction of control (upper) and the invaginating site of adenomyosis (bottom) shown at higher magnification. Scale bars: higher magnification, 20 µm; other, 200 µm. D) Potential EMT‐related ligands from immune ecotypes. Ligand expression levels in sender, and ligand activity, target expression and receptor relative expression in SFRP5^+^ epithelial ecotypes from invaginating microenvironment and the control niche according to NicheNet are shown. E) Cell abundance visualization in spatial coordinates by Cell2location. The junction of the endometrium and myometrium is delineated with a white dashed line. F) Representative immunohistochemical staining images of adenomyosis (AM) and control (UM) showing the expression of CD4 (CD4^+^ T marker), CD8 (CD8^+^ T marker), and CD11C (dendritic cell marker). The junction of the endometrium and myometrium is delineated with a black dashed line. Black triangles indicate the normal endometrium‐myometrium junction (upper) and adenomyosis invaginating site (bottom) shown at higher magnification. Scale bars: higher magnification, 50 µm; other, 500 µm.

In the invaginating microenvironment, SFRP5^+^ epithelial ecotypes exhibited the greatest transcriptional changes between adenomyosis and the control group (Figure 3B). NicheNet^[^ ^37^ ^]^ was used to analyze the activity of ligands received by SFRP5^+^ epithelial ecotypes to disclose the regulatory network. In the invaginating microenvironment, among the ligand signals with the top 50 highest prioritization score received by SFRP5^+^ epithelial ecotypes, numerous ligands have been reported to be associated with EMT (**Figure** **4**A).^[^ ^44^, ^45^, ^46^, ^47^, ^48^, ^49^, ^50^, ^51^, ^52^, ^53^, ^54^, ^55^ ^]^ To further investigate the phenomenon of EMT, we compared the expression of mesenchymal markers in the mesenchymal gene list^[^ ^56^ ^]^ and used mIHC. SFRP5^+^ epithelial ecotypes expressed high levels of mesenchymal markers^[^ ^56^ ^]^ in the invaginating microenvironment (Figure 4B). EMT at the invaginating site was also validated by mIHC: epithelial cells expressed less E‐cadherin but more PDGFR‐α (a stromal fibroblast marker) at the invaginating site of adenomyosis (Figure 4C).

Aside from the typical enhanced distribution of epithelial ecotypes and stromal ecotypes in the invaginating microenvironment, immune ecotypes were unexpectedly enriched at the invaginating site (Figure S5A, Supporting Information). Multiple immune factors have been recognized as inducers of EMT in adenomyosis.^[^ ^29^ ^]^ Using NicheNet, we found some immune‐derived ligands that explained the upregulation of *VIM*, the most significantly up‐regulated mesenchymal gene (Figure 4B), in SFRP5^+^ epithelial ecotypes (Figure 4D). We used Path Explorer in IPA to explore the connection between these immune‐derived ligands and molecules in the gene list “regulation of the epithelial‐mesenchymal transition pathway” of IPA database. TGF beta family, NOTCH family, WNT family, the classic inducers of EMT,^[^ ^57^ ^]^ are involved in the EMT regulation of these immune‐derived ligands (Figure S6A, Supporting Information). In alignment with our results, immune‐derived TNFSF13B (also known as BAFF) and GRN (also known as PGRN) are reported to promote EMT,^[^ ^58^, ^59^ ^]^ and GRN is reported to promote EMT through WNT signaling.^[^ ^60^ ^]^ To investigate the immune subtypes enriched in the invaginating microenvironment, we re‐clustered and classified immune cells in the integrated single‐cell data mentioned above into six subclusters: CD4^+^ T cells (*n* = 371), CD8^+^ T cells (*n* = 478), uterine NK (*n* = 891), peripheral NK (*n* = 90), dendritic cells (DC, *n* = 103), and macrophage (*n* = 271) (Figure S6B–F, Supporting Information), and employed Cell2location^[^ ^25^ ^]^ to show their spatial location. CD4^+^ T cells, CD8^+^ T cells, and DC were enriched at the invaginating site (Figure 4E; Figure S6G, Supporting Information). This occurrence was corroborated by immunohistochemical staining for CD4 (a marker of CD4^+^ T cells), CD8α (a marker of CD8^+^ T cells), and CD11c (a marker of DCs), which showed high protein levels at the invaginating site of adenomyosis (Figure 4F).

In the invaginating microenvironment, SFRP5^+^ epithelial cells underwent EMT in response to microenvironment mediators. Immune cells were concentrated at the invaginating site and may play a role in SFRP5^+^ epithelial cells’ EMT regulation.

### 2.5 ESR1^+^ Smooth Muscle Cells (SMCs) Facilitate Invasion by Creating Migratory Tracts via Collagen Degradation

**Figure 5:**
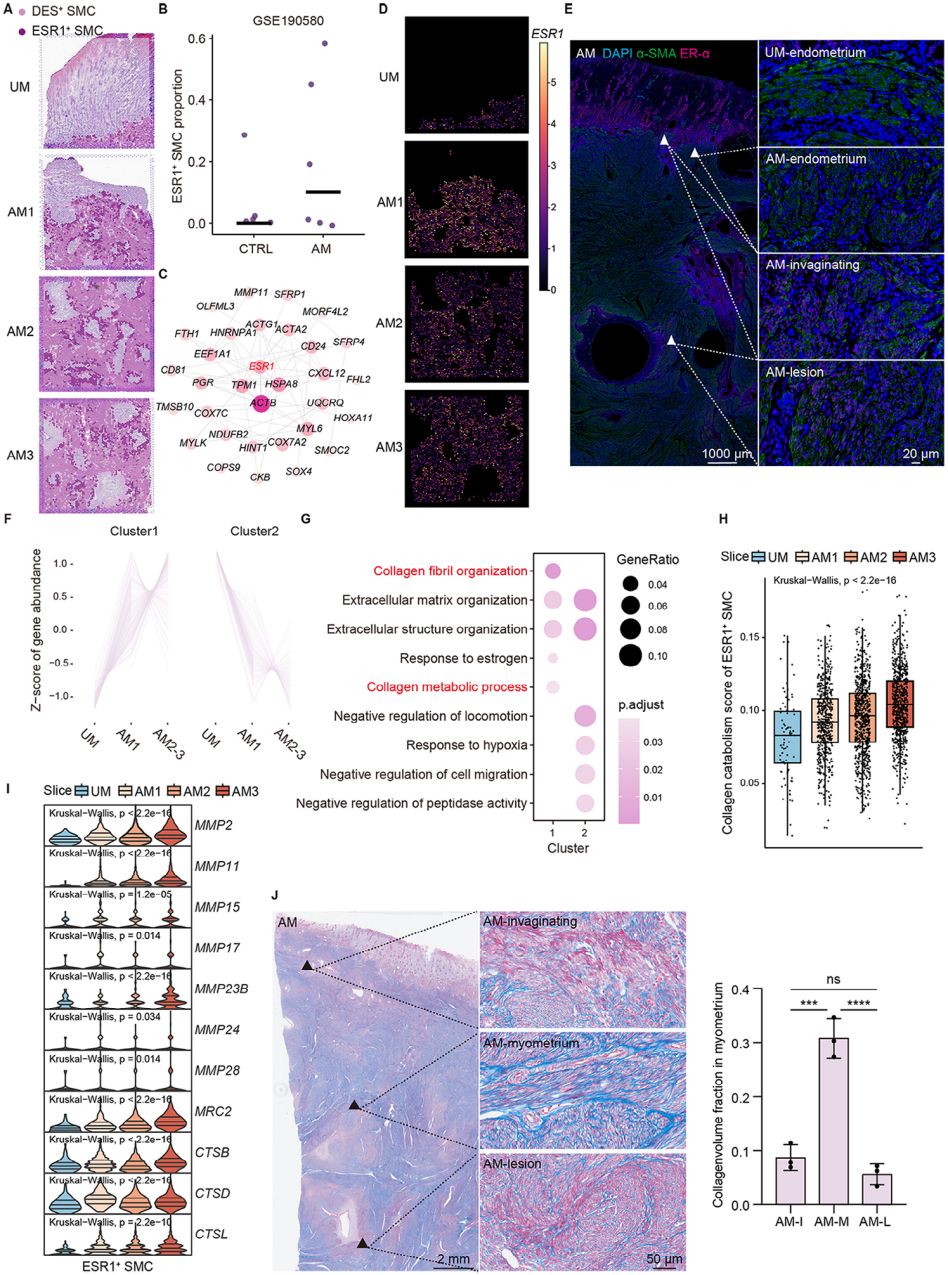
Identification and characterization of SMC sub‐ecotypes. A) Location of two SMC sub‐ecotypes. B) Estimated ESR1^+^ SMC ecotype proportion in bulk RNA‐seq data. C) Molecule interaction network of DEGs in ESR1^+^ SMC ecotypes by STRING. DEGs was obtained by the Wilcoxon test compared to DES^+^ SMC ecotypes. D) Spatial feature plot showing *ESR1* expression across slices. Only SMC ecotypes are shown for better visualization. E) Representative mIHC staining images of adenomyosis showing the expression of α‐SMA (a SMC marker) and ESR1. White triangles indicate myometrium under endometrium (AM‐endometrium), surrounding the invaginating site (AM‐invaginating), and surrounding the lesion (AM‐lesion) of adenomyosis shown at higher magnification. Myometrium under control endometrium (UM‐endometrium) is also shown at higher magnification right upper. Scale bars: left, 1000 µm; right, 20 µm. F‐G) Gene clusters of ESR1^+^ SMC ecotypes with distinct expression patterns in different subgroups by DESeq2, and GOBP terms significantly enriched in each gene cluster. The statistical analysis was performed by Fisher's test. H) Box plot showing collagen catabolism score of ESR1^+^ SMC ecotypes across slices by AUCell. Data are presented as the quartiles. The Kruskal–Wallis rank sum test was used to obtain p‐values. I) Violin plot showing the expression of representative genes of collagen catabolism gene list in ESR1^+^ SMC ecotypes across slices. Data are presented as the quartiles. P‐value was obtained by the Kruskal‐Wallis rank sum test. J) Representative Masson staining images of adenomyosis (left) and collagen volume fraction of Masson staining images in each group (right). Black triangles indicate myometrium surrounding the invaginating site (AM‐invaginating), away from the endometrium and lesion (AM‐myometrium), and surrounding the lesion (AM‐lesion) of adenomyosis shown at higher magnification. Scale bars: higher magnification, 50 µm; other, 2 mm. AM‐I, AM‐invaginating; AM‐M, AM‐myometrium; AM‐L, AM‐lesion. *n* = 3. Data are presented as the mean ± SD. One‐way ANOVA followed by Bonferroni's multiple comparisons test was used to obtain p‐values, ***p < 0.001, ****p < 0.0001.

SMCs are the primary cell type with which ectopic endometrial cells interact during invasion.^[^ ^29^, ^61^ ^]^ To study the role of SMCs in invasion, we re‐clustered and identified two sub‐ecotypes within the SMC ecotypes (Figure S7A,B, Supporting Information). DES^+^ SMC ecotypes (*n* = 7180) showed widespread distribution in the myometrium, while ESR1^+^ SMC ecotypes (*n* = 2176) typically located around the endometrium‐myometrium junction and lesions (**Figure** **5**A; Figure S7C, Supporting Information). The distribution of ESR1^+^ SMC ecotypes indicated their involvement in the invasion process. Deconvolution of bulk RNA‐seq data of adenomyosis myometrium^[^ ^19^ ^]^ exhibited a higher proportion of ESR1^+^ SMC ecotypes within the AM group (Figure 5B), further indicating the importance of ESR1^+^ SMC ecotypes. Differential expression and enrichment analysis showed that ESR1^+^ SMC ecotypes were more likely to respond to estrogen, compared to DES^+^ SMC ecotypes (Figure S7D and Data S22, Supporting Information). Among the genes that were significantly upregulated in ESR1^+^ SMC ecotypes compared to DES^+^ SMC ecotypes (Data S23, Supporting Information), *ESR1* was the core gene in the molecule interaction network (Figure 5C). *ESR1* was highly expressed in the myometrium surrounding the invaginating site and lesions (Figure 5D), indicating that *ESR1* expressed in myometrium might be the spatially specific regulatory molecule of adenomyosis. The presence and location of ERα^+^α‐SMA^+^ cells in mIHC suggested that ESR1^+^ SMCs play a major role in ESR1^+^ SMC ecotypes (Figure 5E). mIHC also demonstrated an increased level of ESR1 in SMCs around invaginating site (AM‐invaginating) and lesion (AM‐lesion) (Figure 5E).

To determine the specific role of ESR1^+^ SMCs, differential gene expression analysis, and Gene Ontology (GO) enrichment analysis were performed on control (slice UM), eutopic endometrium (referred to as slice AM1), and ectopic lesions (referred to as slice AM2 and AM3). The cluster of genes with increased expression from control to lesions (Cluster1 in Figure 5F) was enriched in collagen fibril organization and metabolism (Figure 5F,G; Data S24, Supporting Information). AUCell was employed to determine the collagen catabolism score (the relative expression of collagen catabolism gene set downloaded from GO database) of ESR1^+^ SMC ecotypes. The score significantly increased as the lesions deepened (Figure 5H). Collagen degradation is divided into extracellular and intracellular pathways. Proteolytic enzymes, such as metalloproteinase (MMP) family, participate in the extracellular degradation of collagen. The intracellular pathway involves internalization of collagen and degradation by lysosomal proteases.^[^ ^62^ ^]^ In the collagen catabolism gene set, genes significantly upregulated in ESR1^+^ SMC ecotypes from adenomyosis included members of the MMPs (encoded by *MMP2*, *MMP11*, *MMP15*, *MMP17*, *MMP23B*, *MMP24*, *MMP28*), genes mediating the internalization and lysosomal degradation of collagen (encoded by *MRC2*), and lysosomal proteases (encoded by *CTSB*, *CTSD*, *CTSL*) (Figure 5I). Masson staining confirmed that there was much less collagen in the myometrium around the invaginating site (AM‐invaginating) and the lesions (AM‐lesion) (Figure 5J). These findings suggest that ESR1^+^ SMCs participate in collagen degradation.

To further study the regulatory mechanisms that underpinned the specific biological functions of ESR1^+^ SMCs, we used pySCENIC.^[^ ^63^, ^64^ ^]^ In adenomyosis, the transcription factor *ESR1* was active in ESR1^+^ SMCs (Figure S7E, Supporting Information). To explore the connection among ESR1 regulon and genes related to collagen degradation, IPA Path Explorer was used. In ESR1 regulon, 14 genes had interaction with molecules in gene list of collagen degradation (Figure S7F, Supporting Information). *ESR1* could regulate MMPs (encoded by *MMP1*, *MMP2*, *MMP9*), lysosomal proteases (encoded by *CTSB*, *CTSD*), and member of a disintegrin and metalloprotease domain (ADAM) family (encoded by *ADAM17*). Via molecules in the ESR1 regulon (*FHL2*, *CIRBP*, *IGFBP5*, *RAI14*, *SSBP3*, *PAK4*, *ALCAM*, *RXRA*, *PALLD*, *TNIP1*, *TGFB3*, *HTRA1*, *IGF2*), *ESR1* could also regulate MMPs (encoded by *MMP3*, *MMP7*, *MMP8*, *MMP10*, *MMP11*, *MMP12*, *MMP14*, *MMP15*), lysosomal proteases (encoded by *CTSL*), serine proteases (encoded by *PRSS2*), and convertase (encoded by *FURIN*), collectively forming an interactive regulatory network (Figure S7F, Supporting Information). In line with our results, ESR1 is reported to play a role in collagen degradation,^[^ ^65^ ^]^ and HTRA1 is reported to upregulate matrix MMPs,^[^ ^66^ ^]^ causing collagen degradation.

To summarize, there was a group of SMCs with high expression of transcription factor ESR1 around the invaginating site and lesions of adenomyosis. As structural instability in the myometrium might provide tracks for lesions to invade, ESR1^+^ SMCs may stimulate collagen degradation to form an ectopic endometrial invasion track.

### 2.6 CNN1^+^ Stromal Fibroblasts Are Responsible for Fibrogenesis in Adenomyosis

**Figure 6:**
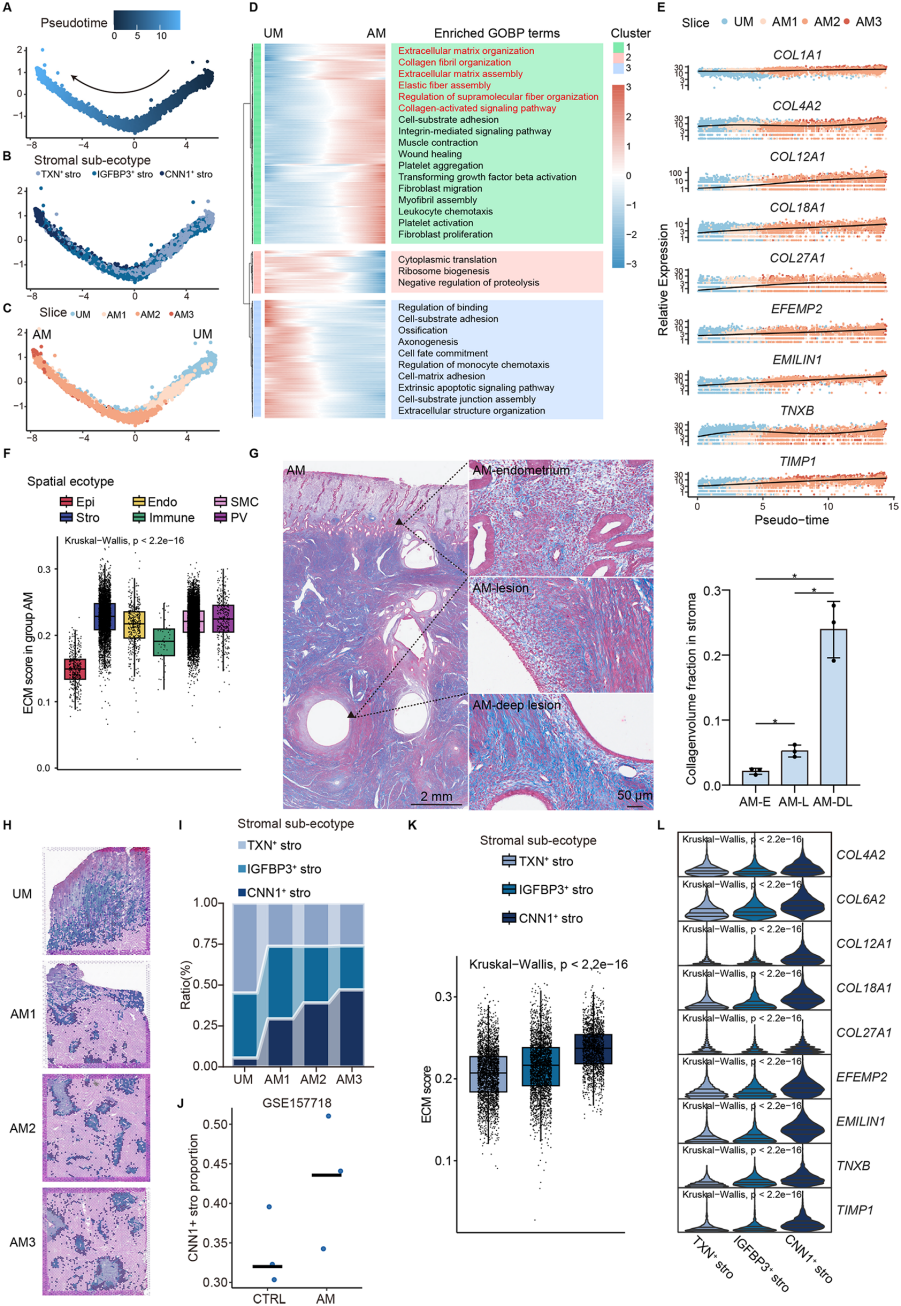
Identification of sub‐ecotypes and trajectory inference within the stromal ecotypes. A–D) Pseudotime trajectory of stromal ecotypes constructed by Monocle. Trajectory colored by pseudotime (A), stromal sub‐ecotypes (B) and slices (C), and GOBP terms significantly enriched in each gene cluster with different pseudo‐temporal expression patterns (D) are shown. The statistical analysis was performed by Fisher's test. E) Expression levels of fibrosis‐related genes along the pseudotime line in (A). Dots are colored by slices. F) Box plot showing ECM score between spatial ecotypes in adenomyosis by AUCell. Data are presented as the quartiles. The Kruskal–Wallis rank sum test was used to obtain p‐values. G) Representative Masson staining images of adenomyosis(left) and collagen volume fraction of Masson staining images in each group (right). Black triangles indicate endometrium at basal layer (AM‐endometrium) and superficial lesion (AM‐lesion) of adenomyosis shown at higher magnification. A deep lesion (AM‐deep lesion) is also shown at a higher magnification right bottom. Scale bars: higher magnification, 50 µm; other, 2 mm. AM‐E, AM‐endometrium; AM‐L, AM‐lesion; AM‐DL, AM‐deep lesion. *n* = 3. Data are presented as the mean ± SD Brown‐Forsythe and Welch's ANOVA tests followed by Dunnett's T3 multiple comparisons test were used to obtain p‐values, *p < 0.05. H) Location of stromal sub‐ecotypes. The color of stromal‐ecotype spots refers to (I). I) Percentage of the three stromal sub‐ecotypes in different slices. J) Estimated CNN1^+^ stromal ecotype proportion in bulk RNA‐seq data. K) Box plot showing ECM score in stromal sub‐ecotypes by AUCell. Data are presented as the quartiles. The Kruskal–Wallis rank sum test was used to obtain p‐values. L) Violin plot showing the expression levels of representative genes involved in fibrosis which are significantly upregulated in CNN1^+^ stromal ecotypes. Data are presented as the quartiles. The Kruskal–Wallis rank sum test was used to obtain p‐values.

The endometrium and adenomyosis lesions are mainly composed of stromal fibroblasts.^[^ ^10^ ^]^ Monocle^[^ ^32^, ^33^, ^34^ ^]^ created the trajectory from normal stroma (UM) to deep‐lesion stroma (AM3), demonstrating the invasion process of stromal fibroblasts from endometrium to deep adenomyosis lesion (**Figure** **6**A–C). Genes involved in fibrosis‐related biological processes, such as ECM organization and collagen fibril organization, were upregulated along the pseudo‐timeline (Figure 6D; Data S25, Supporting Information). Fibrosis‐related genes, such as collagens (encoded by *COL1A1*, *COL4A2*, *COL12A1*, *COL18A1*, *COL27A1*), ECM proteins (encoded by *EFEMP2*, *EMILIN1*, *TNXB*), and matrix MMPs inhibitors (encoded by *TIMP1*), increased in expression level as the lesion deepened (Figure 6E). To demonstrate the relevance of stromal fibroblasts in adenomyosis fibrogenesis, we calculated the ECM and collagen scores (the relative expression of ECM or collagen gene sets calculated by AUCell) of the six spatial ecotypes in adenomyosis and found significantly higher scores in stromal ecotypes (Figure 6F; Figure S8A, Supporting Information). Furthermore, in stromal ecotypes, the two scores increased significantly as the lesion deepened (Figure S8B,C, Supporting Information). Masson staining also showed increased collagen in the stroma as the lesion deepened (Figure 6G). These findings lend credence to the fibrogenesis of the adenomyosis lesion.

To further investigate which stromal sub‐ecotypes played the major role in adenomyosis fibrogenesis, we re‐clustered and classified stromal ecotypes into three sub‐ecotypes based on their gene expression and spatial location (Figure S8D, Supporting Information). TXN^+^ stromal ecotypes (*n* = 2758) were closer to the uterine cavity, IGFBP3^+^ stromal ecotypes (*n* = 2612) were located in the endometrium and the lesion that was close to the myometrium, and CNN1^+^ stromal ecotypes (*n* = 1731) were typically distributed around the invaginating site and lesions (Figure 6H; Figure S8E, Supporting Information), with an increasing ratio as the lesion deepened (Figure 6I). Deconvolution of bulk RNA‐seq data of adenomyosis stromal cells^[^ ^17^ ^]^ showed a higher proportion of CNN1^+^ stromal ecotypes within the AM group (Figure 6J). CNN1^+^ stromal ecotypes showed significantly higher ECM and collagen scores compared to the other stromal sub‐ecotypes (Figure 6K; Figure S9A, Supporting Information). The score increased dramatically in CNN1^+^ stromal ecotypes as the lesion deepened as well (Figure S9B, Supporting Information). In the ECM and collagen gene sets, genes significantly upregulated in CNN1^+^ stromal ecotypes included fibril‐forming collagens (encoded by *COL27A1*), fibril‐associated collagen (encoded by *COL12A1*), collagens network‐forming collagens (encoded by *COL4A2*, *COL6A2*), member of the multiplexin subfamily (encoded by *COL18A1*), ECM proteins (encoded by *EFEMP2*, *EMILIN1*, *TNXB*), and natural inhibitors of the matrix MMPs (encoded by *TIMP1*), compared to the other two stromal sub‐ecotypes (Figure 6L; Data S26, Supporting Information). Given the robust correlation between stromal ecotypes in Visium data and stromal cells in single‐cell data (Figure 1E), we posited that CNN1^+^ stromal fibroblasts played a major role in adenomyosis fibrogenesis.

In conclusion, there was a distinctive distribution of CNN1^+^ stromal fibroblasts around the invaginating site and lesion, with a higher ratio as the lesion deepened. CNN1^+^ stromal fibroblasts performed a crucial role in adenomyosis fibrogenesis.

### 2.7 CNN1^+^ Stromal Fibroblasts Facilitate Fibrosis through Fibroblast‐to‐Myofibroblast Transition (FMT)

**Figure 7:**
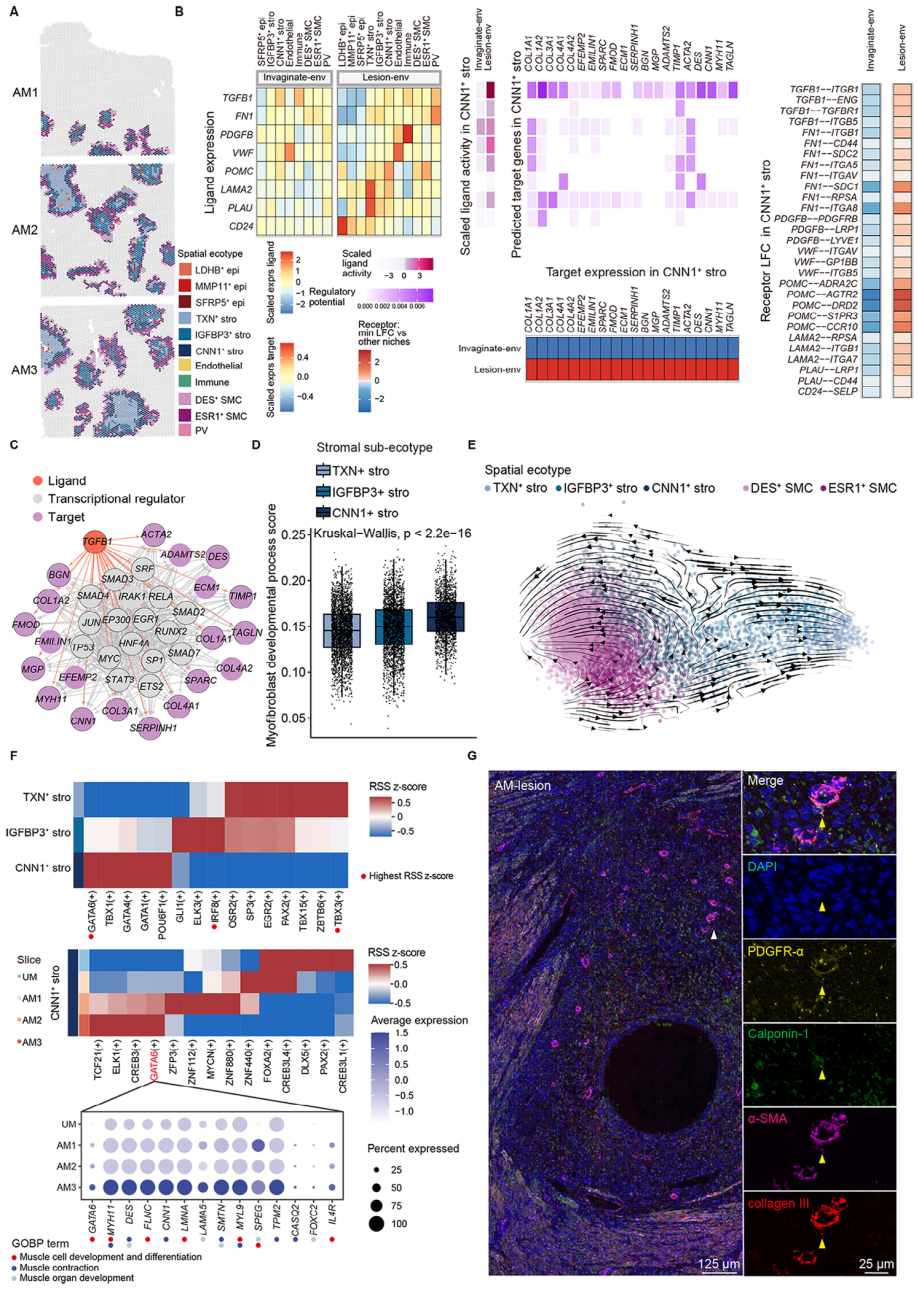
Characterization of CNN1^+^ stromal fibroblasts in lesion microenvironment. A) Components of lesion microenvironment. B) Fibrosis‐related ligand expression levels in sender, and ligand activity, target expression, and receptor relative expression in CNN1^+^ stromal ecotypes from lesion microenvironment according to NicheNet. C) The signaling pathways from *TGFB1* to fibrosis‐related target genes in CNN1^+^ stromal ecotypes inferred by NicheNet. Molecules directly interacted with *TGFB1* are black‐circled and indicated by orange arrows. D) Box plot showing the expression of the gene set about myofibroblast developmental process in each stromal sub‐ecotype by AUCell. Data are presented as the quartiles. The Kruskal‐Wallis rank sum test was used to obtain p‐values. E) RNA velocity of stromal and SMC sub‐ecotypes on a UMAP embedding according to scVelo. Arrows show the direction of movement. F) TF RSS z‐score in stromal sub‐ecotypes (upper) and CNN1^+^ stromal ecotypes across slices (middle) via pySCENIC. The expression of muscle‐related genes in GATA6 regulon in CNN1^+^ stromal ecotypes across slices is shown at the bottom. Regulon with the highest RSS z‐score in each stromal sub‐ecotype is highlighted with a red circle. RSS, regulon specificity score. G) Representative mIHC staining images of adenomyosis lesion (AM‐lesion) showing the expression of PDGFRA (stromal fibroblast marker), CNN1, α‐SMA (SMC marker), and collagen III. White triangles indicate the area shown at higher magnification. Yellow triangles indicate CNN1^+^ stromal fibroblasts undergoing FMT with collagen deposition in an adenomyosis lesion. Scale bars: left, 125 µm; right, 25 µm.

Fibrosis is the primary cause of adenomyosis development and accounts for the ineffective treatment in clinical practices.^[^ ^26^ ^]^ To investigate the regulatory mechanism of CNN1^+^ stromal fibroblasts in fibrosis, we analyzed cell–cell interaction in the lesion microenvironment (lesion‐env, as shown in **Figures** **7**A and S9C, Supporting Information) by NicheNet.^[^ ^37^ ^]^ We found some prioritized ligands that explained the upregulation of fibrosis‐related target genes in CNN1^+^ stromal ecotypes (Figure 7B). In the lesion microenvironment, ligand‐receptor pairs containing *TGFB1*, *FN1*, *PDGFB*, *VWF*, *LAMA2*, *PLAU*, and *CD24* exhibited strong interaction (Figure S9D, Supporting Information), according to CellPhoneDB.^[^ ^67^ ^]^ We further explored the signaling pathways among these prioritized ligands and fibrosis‐related target genes by NicheNet (Figure 7C; Figure S9E, Supporting Information). TGFβ is a potent regulator of fibrosis.^[^ ^68^ ^]^ As one of the three isoforms of TGFβ, TGFB1 has a critical role in the progression of fibrosis.^[^ ^69^, ^70^ ^]^ We found 17 transcriptional regulators connecting TGFB1 signal and alteration of fibrosis‐related genes in CNN1^+^ stromal ecotypes (Figure 7C), which included regulators in the canonical SMAD pathway.^[^ ^69^ ^]^ Classically, activated SMAD2/SMAD3 complexes with SMAD4 to regulate transcription of profibrogenic genes.^[^ ^68^ ^]^ Other fibrosis‐related ligands in lesion microenvironment, such as *FN1*, *PDGFB*, *VWF*, *POMC*, *LAMA2*, *PLAU* and *CD24*, could also regulate expression of these profibrogenic target genes directly or indirectly through SMAD (Figure S9E, Supporting Information). These findings suggested that mediators in the lesion microenvironment facilitated the fibrogenesis activity of CNN1^+^ stromal fibroblasts.

Fibroblast‐to‐myofibroblast transition (FMT) is an important process of fibrosis.^[^ ^71^, ^72^ ^]^ CNN1^+^ stromal ecotypes, unlike other stromal ecotypes, had higher expression of genes related to ECM assembly and muscle development (Figure S10A and Data S27, Supporting Information). To investigate whether CNN1^+^ stromal ecotypes undergo FMT, we further conducted AUCell scoring and RNA velocity^[^ ^73^ ^]^ analysis. CNN1^+^ stromal ecotypes exhibited significantly elevated score in gene set associated with the myofibroblast developmental process in comparison to other stromal sub‐ecotypes (Figure 7D). The score exhibited a significantly increase in CNN1^+^ stromal ecotypes as the lesion progressed in depth (Figure S10B, Supporting Information). Moreover, *ACTA2* (encoding α‐SMA), the myofibroblast‐associated genes,^[^ ^70^ ^]^ showed distinct high level in CNN1^+^ stromal ecotypes (Figure S10C, Supporting Information). As α‐SMA was employed as both a myofibroblast marker and an SMC marker,^[^ ^74^ ^]^ myofibroblast might be grouped into the SMC ecotypes by unsupervised clustering. We evaluated RNA velocity^[^ ^73^ ^]^ in SMC sub‐ecotypes and stromal sub‐ecotypes to further determine whether FMT occurred in adenomyosis and which sub‐ecotypes was mainly responsible. CNN1^+^ stromal ecotypes were undergoing FMT, with the transition direction toward an SMC‐like expression pattern (Figure 7E; Figure S10D, Supporting Information). We used pySCENIC^[^ ^63^, ^64^ ^]^ to investigate the regulatory mechanism of FMT in CNN1^+^ stromal fibroblasts. Compared to the other two stromal sub‐ecotypes, *GATA6* was the transcription factor with the highest regulon specificity (RSS) score (z‐score = 0.4864) in CNN1^+^ stromal ecotypes (upper of Figure 7F; Data S28, Supporting Information). As the lesion deepened, the transcription activity of *GATA6* was increased in CNN1^+^ stromal ecotypes (middle of Figure 7F; Data S29, Supporting Information). In GATA6 regulon, numerous genes were related to muscle cell development and differentiation, muscle contraction and muscle organ development (bottom of Figure 7F). Compared to fibroblasts, myofibroblasts are structurally and functionally close to smooth muscle.^[^ ^70^ ^]^ *GATA6* may mediate the acquisition of this muscle‐related characteristic in FMT of CNN1^+^ stromal fibroblasts. The marker of CNN1^+^ stromal ecotypes (*CNN1*), the marker of FMT (*ACTA2*), and the markers of ECM deposition (*COL6A2*, *COL12A1*, *COL18A1*, *EMILIN1*, and *TIMP1*) showed the same spatial expression pattern (Figure S10E–K, Supporting Information). For validation, we used mIHC labeling PDGFR‐α, Calponin‐1 (protein of *CNN1*), α‐SMA, and collagen III (a marker of fibrosis^[^ ^75^ ^]^). Colocalization of PDGFR‐α and Calponin‐1 indicated the existence of CNN1^+^ stromal fibroblast, while colocalization of PDGFR‐α and α‐SMA validated FMT. Collagen III indicated collagen deposition in CNN1^+^ stromal fibroblasts (Figure 7G).

Overall, cell–cell communication in the lesion microenvironment facilitated the pro‐fibrotic function of CNN1^+^ stromal fibroblasts. Under the regulation of transcription factor *GATA6*, CNN1^+^ stromal fibroblasts induced fibrogenesis via FMT.

## 3 Discussion

The limited understanding of adenomyosis's natural history significantly hinders the progression of effective clinical treatments.^[^ ^76^ ^]^ Our study utilized spatial transcriptome technology in combination with three existing single‐cell datasets^[^ ^12^, ^23^, ^24^ ^]^ to provide a comprehensive overview of spatial transcriptional features and cellular interactions from the invaginating site to deep lesions of adenomyosis. We investigated the molecular features of invagination, invasion, and fibrosis by delineating a detailed transcriptional map using spatial transcriptomics, and discovered the predominant subpopulations responsible for these three processes, presenting new insights for future study and the development of diagnostic and therapeutic strategies.

Recent advances in adenomyosis pathogenesis highlight three interconnected pathological axes: endometrial invagination driven by basal endometrium dysfunction, estrogen‐mediated microenvironment remodeling, and fibrotic niche consolidation.^[^ ^7^, ^26^ ^]^ Our spatial transcriptomic findings bridge these axes by revealing their spatial coordination. The SFRP5^+^ epithelial cells at invagination sites secrete IHH that may establish a pro‐invasive microenvironment, while CNN1^+^ stromal fibroblasts in deep lesions exhibit fibrosis‐specific markers. This spatial progression from invagination to fibrosis aligns with the tissue injury and repair theory, where repeated microtrauma at the endometrial–myometrial interface initiates a self‐sustaining cycle of invasion and fibrotic repair.^[^ ^26^ ^]^

The invagination hypothesis is largely acknowledged as the basic etiological theory for adenomyosis. Targeted deep sequencing of adenomyosis lesions and adjacent basalis endometrial glands demonstrated that epithelial cells in endometrial basalis may induce invagination.^[^ ^7^, ^8^, ^77^ ^]^ However, the specific cause of basalis endometrial invagination, the alteration in gene expression in epithelial cells, and the signaling network with other cells during the invagination process remain unknown. We identified a group of SFRP5^+^ epithelial cells that might take part in adenomyosis pathogenesis and found cellular interactions via IHH, secreted by SFRP5^+^ epithelial, were significantly enhanced in the invaginating microenvironment, promoting endometrium proliferation and angiogenesis through autocrine and paracrine. Previous studies also demonstrated that the Hedgehog signaling pathway stimulated the proliferation of epithelial cells^[^ ^41^ ^]^ and stromal fibroblasts,^[^ ^42^ ^]^ as well as angiogenesis.^[^ ^43^ ^]^ Furthermore, we also found that *IHH* might promote EMT of SFRP5^+^ epithelial cells in conjunction with other mediators in the invaginating microenvironment. Hedgehog signaling is one of several canonical signaling pathways to induce transcription program switching in EMT.^[^ ^78^ ^]^ Immune cells may also play a role in the regulation of SFRP5^+^ epithelial cells’ EMT process based on their distribution in the invaginating microenvironment. Consistent with our study, IHH exhibited disease‐related expression in endometriosis. Lawrenson et al. reported a group of IHH^+^ epithelial cells detected in endometriosis lesions but absent from eutopic endometrium in endometriosis patients.^[^ ^79^ ^]^ However, Li et al. reported decreased IHH signaling in adenomyosis eutopic endometrial tissues (from adenomyosis patients who underwent a hysterectomy, which might include endometrial basalis) compared to leiomyoma endometrial tissues (from patients who undergoing laparoscopic myomectomy, endometrial basalis should be retained for fertility).^[^ ^80^ ^]^ The argument could be attributable to variances in the endometrial samples collected, as we only gathered samples with typical invaginating structures of adenomyosis and the sections from both groups contained a full layer of endometrium. We observed that the IHH level was higher in sections with apparent invaginating structure than in sections without this feature. Despite this, Li et al. reported increased IHH signaling levels in the functional layer in both groups compared to the endometrial basalis,^[^ ^80^ ^]^ which was consistent with our results. Additionally, spatial transcriptomics revealed higher expression of *IHH* in SFRP5^+^ epithelial cells which were distributed in endometrial basalis in adenomyosis, which would have been hidden in bulk estimation. Further studies are needed to determine the precise involvement of IHH in adenomyosis pathogenesis.

Previous studies have focused on the functions of epithelial cells and stromal fibroblasts, the two primary components of endometrium, in the invasive process of adenomyosis. The roles of SMCs, the major cell types in the myometrium, remain largely unknown. Using spatial transcriptomics, we identified a unique set of ESR1^+^ SMCs that were specifically distributed around the invaginating sites and ectopic lesions. ESR1^+^ SMCs were associated with collagen degradation, thus facilitating the formation of the track for ectopic endometrial invasion. ERα‐/‐ animals had considerably higher collagen content in their skin, indicating a link between elevated ERα levels and decreased collagen deposition.^[^ ^65^ ^]^ Li et al. also found higher ESR1 levels in endometrium‐myometrium junction SMCs in the adenomyosis group, which mediated estrogen‐induced aberrant myometrial peristalsis.^[^ ^81^ ^]^ Adenomyosis is an estrogen‐dependent disease, and estrogen is involved in a variety of biological processes related to adenomyosis pathogenesis.^[^ ^6^, ^82^ ^]^ Previous studies have shown the significance of ERα in adenomyosis pathogenesis. Tamoxifen, a selective ERα modulator that has been reported to activate nuclear ERα in SMCs,^[^ ^83^ ^]^ was linked to a high frequency of adenomyosis in breast cancer patients,^[^ ^84^, ^85^ ^]^ and was used to generate adenomyosis mouse models.^[^ ^86^ ^]^ Besides, ERα was linked to the loss of noradrenergic nerve fibers in adenomyosis.^[^ ^87^ ^]^ In the realm of adenomyosis pathogenesis, the estrogen receptor beta (ERβ), encoded by the *ESR2* gene, has been a focus of greater scrutiny compared to ERα. This is primarily attributed to two key factors. First, the expression of ESR2 in the glands, stroma, and myometrium of adenomyosis is generally elevated compared to normal uterus, whereas ESR1 shows variations during distinct menstrual cycle phases and tissue types.^[^ ^88^ ^]^ Second, ERα and ERβ promote ectopic foci proliferation and inflammation in the pathogenesis of endometriosis, while ERβ promotes ectopic lesions’ survival through anti‐apoptosis, inflammasome activation, and invagination.^[^ ^82^, ^89^ ^]^ With spatial transcriptomics, ERα was identified for the first time as a spatially specific regulatory molecule in SMCs in adenomyosis in our study.

Fibrosis is a major contributor to the resistance to hormonal treatment in adenomyosis,^[^ ^90^ ^]^ and it is intimately linked to adenomyosis‐induced dysmenorrhea.^[^ ^91^ ^]^ Myofibroblasts are the main effector cells of fibrosis,^[^ ^72^, ^92^ ^]^ resulting from FMT that is triggered by profibrotic mediators.^[^ ^71^, ^74^ ^]^ We found that CNN1^+^ stromal fibroblasts played an important role in fibrogenesis of adenomyosis. CNN1^+^ stromal fibroblasts expressed high levels of fibrosis markers in response to ligand signaling from the lesion microenvironment and underwent FMT via the regulation of *GATA6*, which promoted fibrogenesis. Consistent with our result, GATA6 was reported to show higher expression levels in stromal fibroblasts of adenomyosis.^[^ ^93^ ^]^ Additionally, high GATA6 expression might cause tracheal fibrosis by triggering fibroblast activation.^[^ ^94^ ^]^ In the lesion microenvironment, the mediators produced by epithelial ecotypes, stromal ecotypes, endothelial ecotypes, immune ecotypes, SMC ecotypes, and PV ecotypes worked together to form a profibrotic environment. Consistently, Guo et al. reported that TGF‐β/Smad signaling drove FMT and fibrosis in adenomyosis, which has been verified in both human^[^ ^95^ ^]^ and mice.^[^ ^96^ ^]^ We identified CNN1^+^ stromal fibroblasts as the major cell types responsible for fibrogenesis in adenomyosis and identified the traits of the profibrotic microenvironment.

Currently, the predominant medical approach to adenomyosis management involves the use of hormonal therapies designed to induce a hypoestrogenic state.^[^ ^4^, ^97^ ^]^ However, hormonal treatments only provide short‐term symptom relief, accompanied by unwanted side effects.^[^ ^26^, ^76^ ^]^ The identification of molecules that play a dominant role in the main processes of adenomyosis pathogenesis could contribute to more precise and targeted therapies. Microarray^[^ ^14^, ^15^ ^]^ and RNA sequencing^[^ ^16^, ^17^, ^18^, ^19^ ^]^ studies highlighted the significant changes in processes such as EMT, inflammation, ECM organization, steroid hormone response, and angiogenesis. Shi et al. performed single‐cell RNA sequencing of eutopic endometrium and ectopic lesion from adenomyosis in secretory phase, revealing a unique population of lesion‐specific cells. These cells exhibited high‐level copy number changes and were undergoing epithelial‐endothelial transformation.^[^ ^12^ ^]^ Bulun et al. conducted single‐cell RNA sequencing analyses on samples from the endometrium, lesions, and myometrium of proliferative adenomyosis tissues. This study identified a distinct group of stromal fibroblasts within the lesions that interacted with epithelial cells through the WNT/SFRP signaling pathway.^[^ ^10^ ^]^ Spatial transcriptomics is a powerful tool to enable the resolution of spatially characterized gene expression and cellular interactions.^[^ ^21^, ^22^, ^98^ ^]^ Spatial transcriptomics enabled the visualization of spatial distribution for unique adenomyosis‐related subpopulations discovered using single‐cell RNA sequencing. Che et al. used single‐cell analysis in combination with spatial transcriptomics and identified the unique cell subpopulations with stem cell or progenitor cell properties as well as the invasive cell subpopulations within adenomyosis lesions.^[^ ^11^ ^]^ Our study is the first omics study focused on the penetrating process of ectopic endometrium in adenomyosis with typical invagination features. We used spatial transcriptomics in conjunction with single‐cell RNA sequencing to investigate the molecular mechanisms underlying invagination at the endometrial–myometrial interface, inside‐to‐outside invasion, and lesion fibrosis, the three important pathological processes of invagination‐related adenomyosis to fulfill our understanding of the pathogenesis of adenomyosis from another aspect. We identified unique targets with spatial distribution for possible drug discovery that targets the key processes in adenomyosis.

The identification of spatial and phase‐specific therapeutic targets provides actionable strategies for precision management. Early detection: The IHH secreted by SFRP5^+^ epithelial cells at invagination sites, combined with the EMT‐promoting factors they receive, creates a unique pro‐invagination microenvironment, offering potential biomarkers for liquid biopsy. Marker‐carrying exosomes isolated from uterine cavity fluid could serve as a minimally invasive diagnostic tool, particularly when combined with ultrasound detection of junctional zone abnormalities. Invasion interception: The spatially restricted ESR1^+^ SMCs surrounding early lesions present an ideal target for proteolysis targeting chimera (PROTAC)‐based therapies. ERα degraders (e.g., ARV‐471^[^ ^99^ ^]^) could be locally delivered via intrauterine sustained‐release systems, selectively eliminating collagenolytic SMCs with muscle tissue specific E3 ligases,^[^ ^100^ ^]^ while preserving normal myometrial function. This approach capitalizes on the spatial specificity of ESR1^+^ SMCs to avoid systemic estrogen suppression. Fibrosis reversal: The dissection of the pro‐fibrotic microenvironment in deep lesions offers potential molecular targets for the development of targeted anti‐fibrotic therapies. Target inhibitors with myometrium‐penetrating nanoparticles could disrupt the fibrotic cascade. The enhanced permeability and retention effect in fibrotic lesions would ensure targeted drug accumulation, potentially reversing established fibrosis without affecting normal stroma.

Our study provides a comprehensive transcriptional map from the invaginating site to deep lesions in adenomyosis, however, there are still several shortcomings that must be addressed. While we validated the relevance of core spatial ecotypes to adenomyosis through bulk RNA‐seq data deconvolution and histological methods, it is essential to conduct further large‐scale studies to explore the correlation between these spatial ecotypes (as well as the histologically focused core cell types) and the diverse clinical manifestations of adenomyosis, given the limitation of our limited sample size. Additionally, as one spot of Visium platform contains multiple cells, we would lose the information of cell types with sparse distribution, such as endometrial stem cells. Metaplasia is another major theory for the etiology of adenomyosis, emphasizing the role of endometrial stem cells in pathogenesis.^[^ ^5^, ^11^ ^]^ Previous studies have shown multiple lines of evidence supporting the metaplasia theory.^[^ ^11^, ^101^ ^]^ Besides, some cell types may be shadowed by other ecotypes because of the low quantity in each spot, such as immune cells and PVs, leading to limited number of DEGs. Technologies such as single‐cell or sub‐cell resolution spatial transcriptome technologies^[^ ^22^ ^]^ are more suited to study the spatial characteristics of these sparsely distributed cell types.

**Figure 8:**
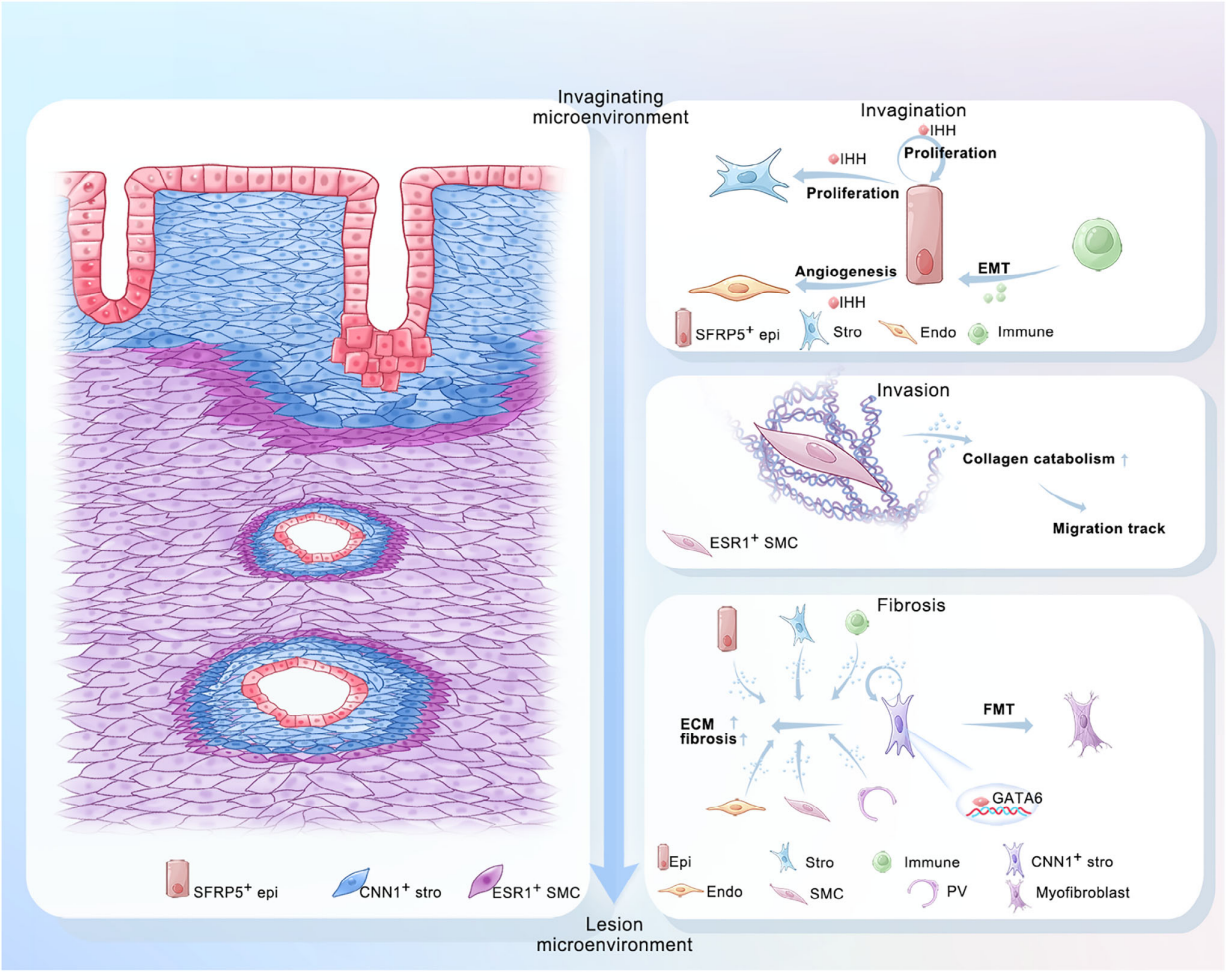
Schematic representation of major participants and molecular pathogenesis in the progression of invagination‐related adenomyosis. Based on spatial transcriptomics, we revealed the molecular features of three main processes in invagination‐related adenomyosis pathogenesis from invaginating site to deep lesion. SFRP5^+^ epithelial cells, ESR1^+^ SMCs, and CNN1^+^ stromal fibroblasts were responsible for invagination, invasion, and fibrosis in adenomyosis progression, respectively. We elucidated the detailed mechanism of cell interactions and microenvironment mediators, providing new insight for further target exploration.

Our spatial mapping reconciles longstanding debates between invagination and metaplasia theories. While SFRP5^+^ epithelial invagination initiates lesion formation (supporting the invagination theory), subsequent transition features in deep lesions suggest metaplastic transformation may sustain lesion survival. This spatiotemporal evolution implies that therapeutic strategies should adapt to disease phases: invagination inhibitors for early‐stage prevention versus anti‐fibrotic agents for advanced disease. In conclusion, we generated a comprehensive spatial transcriptional profile of adenomyosis from the invaginating site to deep lesions, identifying key molecules involved in invagination, invasion, and fibrosis (**Figure** **8**). We identified the novel targets for further study and possible drug discovery that target the main processes involved in adenomyosis progression. Furthermore, we established the first transcriptome web resource for adenomyosis, which will be a useful resource for future studies.

## 4 Experimental Section

### Human Samples

The study was under the approval of local medical ethics from Renji Hospital, Affiliated with Shanghai Jiao Tong University School of Medicine (No. KY2021‐211‐B). The study collected fresh samples from 16 patients who underwent hysterectomy for adenomyosis and 11 control patients with uterine fibroids (Data S1, Supporting Information). The inclusion criteria included: age less than 50 years old; regular menstruation; normal Body Mass Index; no history of smoking; no record of infectious, malignant, and immune diseases; no use of hormones or intrauterine devices in the past three months; without endometriosis, hydrosalpinx or other reproductive system diseases. Part of the fresh samples was embedded in an optimal cutting temperature compound (Sakura, #4583), transported in dry ice, and stored at −80 °C. The rest was fixed in 4% paraformaldehyde and then embedded in paraffin. All patients signed informed consent.

### Spatial Transcriptomics and Data Processing

Spatial transcriptomic sequencing was performed on one section (UM) far away from the leiomyoma site of the fibroid patient and three layer‐by‐layer 6.5mm × 6.5 mm sections (AM1, AM2, AM3) from the invaginating site to deep lesions of an adenomyosis sample with typical invagination features using the 10x Visium platform. The Space Ranger software pipeline (version 1.2.0; https://www.10xgenomics.com/support/software/space‐ranger/latest) was used to process Visium Spatial Gene Expression data with brightfield microscope images, aligning to GRCh38 genome. The Seurat^[^ ^102^ ^]^ package (version 4.3.0) was used to read the output of the Space Ranger pipeline and used SCTransform^[^ ^103^ ^]^ to normalize the data, and the mutual nearest neighbors algorithm^[^ ^104^ ^]^ to eliminate batch effects.

### Integration with Single‐Cell Data

First, single‐cell data were used from three published databases^[^ ^12^, ^23^, ^24^ ^]^ to construct gene expression patterns of six common cell types of uterus endometrium and myometrium: epithelial cells, stromal fibroblasts, endothelial cells, immune cells, SMCs, and PV. After downloading the raw data of single‐cell RNA sequencing, the lessons from Harvard Chan Bioinformatics Core (https://hbctraining.github.io/scRNA‐seq_online/schedule/links‐to‐lessons.html) were followed to filter, normalize, integrate, cluster, and define these single‐cell data. Cells with the number of UMIs less than 500, or the number of genes detected less than 250, or the novelty score (divide the log10 number of genes by the log10 number of UMIs) less than 0.8, or the proportion of transcripts mapping to mitochondrial genes more than 0.2 were filtered. Genes which were expressed in less than 10 cells were filtered. The SCTransform^[^ ^103^ ^]^ method was used to normalize the data. The CCA was used for integration. Second, Cell2location^[^ ^25^ ^]^ (version 0.1.3) was used to integrate spatial transcriptome data with single‐cell data. Negative binomial regression was employed to estimate reference cell type signatures with the default parameters of gene filtering. Cell2location spatial mapping model was used to quantify individual cell type abundance with the following model hyper‐parameters: N_cells_per_location = 30, detection_alpha = 20. Then the cell type with the highest proportion was utilized to annotate corresponding spatial spots. Third, Pearson correlation analysis was performed between the spatial transcriptome data and the single‐cell data on the expression level of DEG of each cell type. As for the spatial location of immune cells, single‐cell data annotated as immune were re‐clustered and classified into six main groups based on marker expression: *CD3G* for CD4^+^ T cells; *CD8A* for CD8^+^ T cells; *NKG7* for NK; *CD1C* for DC; *CD14* and *CD163* for macrophage. NK was further divided into uterine NK and peripheral NK according to the expression of *ITGA1*(also known as *CD49A*, the tissue‐resident marker),^[^ ^105^ ^]^ *FCGR3A* (also known as *CD16*, peripheral NK marker),^[^ ^29^ ^]^ and *B3GAT1*(also known as *CD57*, peripheral NK marker).^[^ ^29^ ^]^ Cell2location^[^ ^25^ ^]^ was also employed to map subclusters of immune cells using the same parameters, with sub‐immune cells as well as other five cell types as input single‐cell data.

### Re‐Cluster Analysis of the Spatial Ecotypes

To delve deeper into the dysregulation of the endometrium and myometrium, epithelial ecotypes, stromal ecotypes, and SMC ecotypes were re‐clustered. The spots annotated as “epithelial ecotypes”, “stromal ecotypes” and “SMC ecotypes” were extracted, normalized by SCTransform^[^ ^103^ ^]^ method, integrated by the CCA, and clustered based on principal components. The elbow plot in Seurat^[^ ^102^ ^]^ package was used to determine the number of principal components used for clustering. Each sub‐ecotype was annotated based on the important signaling enriched and genes with high expression level considering commercially available antibodies for further clarification.

### Deconvolution Analysis of Bulk RNA‐seq Data

To further estimate the association of core spatial ecotypes with adenomyosis, MuSiC2 (version 1.0.0) was used for deconvolution.^[^ ^31^ ^]^ Bulk RNA sequencing expression data of adenomyosis and control was downloaded from the Gene Expression Omnibus (GEO) database (Accession No. GSE244236, GSE157718, GSE190580). The tutorials of MuSiC2 with defaulted parameters were followed and cell type specific DEGs were detected using T statistics.

### Differential Gene Expression and Enrichment Analysis

To compare the overall differences between adenomyosis and control patients in each spatial ecotype, AM1, AM2, and AM3 were named as group AM, while UM as group UM, and Deseq2(version 1.40.2) was used to perform pseudo‐bulk differential expression analysis, following the lessons from Harvard Chan Bioinformatics Core (https://hbctraining.github.io/scRNA‐seq_online/lessons/pseudobulk_DESeq2_scrnaseq.html). Similarly, Deseq2 was employed for differential expression analysis on normal endometrium (UM), eutopic endometrium of adenomyosis (AM1), and ectopic endometrium (AM2 and AM3). To compare the differences between different samples or sub‐ecotypes, the “FindAllMarkers” function of the Seurat was used with the following parameters: min.pct = 0.25, thresh.use = 0.25. The GO knowledgebase (https://www.geneontology.org/) and IPA (content version 107 193 442) were employed to conduct an enrichment analysis of biological functions and signaling pathways. To investigate the gene expression differences between invaginate‐env and UM‐niche, the “FindMarkers” function of the Seurat (parameters: min.pct = 0.25, thresh.use = 0.25) and the “jjVolcano” function of scRNAtoolVis (version 0.0.5; https://junjunlab.github.io/scRNAtoolVis‐manual/index.html) were used to visualize DEG (adjusted*p*‐value < 0.05).

### Pseudo‐Time Analysis

To explore the changes of epithelial ecotypes and stromal ecotypes, and investigate the impact of spatial location on gene expression, Monocle^[^ ^32^, ^33^, ^34^ ^]^(version 2.28.0) was used to construct trajectories. The tutorials of Monocle were followed to load up data into CellDataSet, estimate size factors and dispersions, filter low‐quality spots (parameters: min_expr = 0.1, num_cells_expressed ≥ 10), order spots in pseudotime and cluster genes by pseudotemporal expression pattern. Then the GO knowledgebase was employed to conduct enrichment analysis on pseudotemporal specific genes. Benjamini–Hochberg correction was used to adjust the P value. Significant ontologies were determined by a q‐value cutoff of 0.05. To discover the genes that act as on/off switches along the epithelial pseudotime line, and the ordering at which these switches occur, GeneSwitches (version 0.1.0; https://geneswitches.ddnetbio.com/) was used to discover the order of switching genes during cell state transitions after Monocle. The tutorials of GeneSwitches were followed to convert Monocle results into SingleCellExperiment objects, binarize gene expression, fit logistic regression, estimate switching time, and visualize the ordering of switching genes. To study FMT in the lesion microenvironment, RNA velocity analysis was performed via scVelo^[^ ^73^ ^]^ (version 0.2.5), which solved the full transcriptional dynamics of splicing kinetics, to infer cell trajectories. The “RNA Velocity Basics” tutorials were followed to preprocess the data of stromal ecotypes and SMC ecotypes, estimate RNA velocity, and project the velocities.

### Cell Communication Analysis

To explore spatially related cell–cell interactions, stLearn^[^ ^36^ ^]^ (version 0.4.7) was used to find areas with high cell–cell interactions. The spots in highly interacting areas were extracted and the statistical analysis method of CellphoneDB^[^ ^67^ ^]^ (version 4.1.0) was used to analyze differentially expressed ligand‐receptor pairs. Further, NicheNet^[^ ^37^ ^]^ (version 1.1.1) was used to explore the specific interactions between different spatial ecotypes in the invaginating microenvironment and lesion microenvironment. The “Differential NicheNet analysis between niches of interest” tutorials was followed to define the niches of interest (invaginate‐env vs UM‐niche; lesion‐env vs invaginate‐env), calculate differential expression between the niches, calculate ligand activities and infer active ligand‐target links, calculate expression of ligands, receptors and targets across ecotypes of interest, score ligand‐receptor interactions and prioritize ligand–receptor–target links (parameters: “scaled_ligand_score_spatial” = 0, other as default). The “Inferring ligand‐to‐target signaling paths” tutorials of NicheNet was followed to infer signaling paths between ligands and target genes of interest with default parameters.

### Molecule Interaction Network Analysis

To investigate the relationship between Hedgehog signaling and enriched biological processes in the invaginating microenvironment, the “FindAllMarkers” function of the Seurat (parameters: min.pct = 0.25, thresh.use = 0.25) was used to find the DEGs (adjusted *p*‐value < 0.05), used STRING^[^ ^106^ ^]^ (version 12.0) to construct the interaction network of the corresponding proteins and used Cytoscape (version 3.9.1) for visualization. The GO knowledgebase was employed to obtain enriched biological processes. Benjamini–Hochberg correction was used to adjust the P value. Significant ontologies were determined by a q‐value cutoff of 0.05. Genes involved in the Hedgehog signaling pathway were downloaded from the Kyoto Encyclopedia of Genes and Genomes database (https://www.genome.jp/kegg/). To investigate the relationship between indicated immune‐derived ligands and EMT, as well as the relationship between ESR1 and collagen‐degradation genes, Path Explorer and Connect function in IPA were used. EMT‐related and Collagen‐degradation related molecules were downloaded from ingenuity canonical pathway of IPA using annotation “regulation of the epithelial‐mesenchymal trasition pathway” and “collagen degradation”, respectively.

### Gene Set Score

AUCell (https://bioconductor.org/packages/release/bioc/vignettes/AUCell/inst/doc/AUCell.html; version 1.22.0) was employed to calculate the relative expression of genes related to Hedgehog signaling pathway, collagen catabolism, ECM, collagen, and developmental process of myofibroblast using the “Area Under the Curve”. Gene set of Hedgehog signaling pathway was downloaded from Kyoto Encyclopedia of Genes and Genomes Database (https://www.genome.jp/kegg/) using identifier hsa04340. Gene sets of collagen catabolism, ECM, and collagen were downloaded from the GO resource (https://www.geneontology.org/) using annotation “collagen catabolic process”, “extracellular matrix structural constituent”, and “collagen fibril organization”, respectively. Gene set of developmental process of myofibroblast was downloaded from the IPA using annotation “developmental process of myofibroblasts”. The AUCell tutorial (https://bioconductor.org/packages/release/bioc/vignettes/AUCell/inst/doc/AUCell.html) was followed to score gene signatures and used “ggboxplot” in R package ggpubr (version 0.6.0; https://rpkgs.datanovia.com/ggpubr/) for visualization.

### Transcription Factor Regulon Analysis

pySCENIC^[^ ^63^, ^64^ ^]^ (version 0.12.1) was used to infer transcription factors in gene regulatory networks. The pySCENIC tutorial was followed to infer co‐expression modules, prune indirect targets, quantify the activity of regulons, and calculate RSS. z‐score was utilized for the heatmap visualization of RSS. The regulon enrichment of pySCENIC and the DEG screened by “FindAllMarkers” were combined to determine the significance of a regulon.

### Cell Culture

The Ishikawa cell line (BNCC, BNCC354856) was used as a cell model for endometrial epithelial cells, an ihESC (the American Type Culture Collection, CRL‐4003) for stromal fibroblasts, and HUVEC (BNCC, BNCC342438) for endothelial cells. They were cultured in DMEM/F‐12 (1:1) medium without phenol red (BasalMedia, L340KJ) with 10% certified fetal bovine serum charcoal‐stripped (Lumiprobe, 04‐201‐1A) and 1% Penicillin–Streptomycin–Neomycin antibiotic mixture (Gibco, 15 640 055) at 37 °C in a humidified 5% CO_2_ incubator.

### Cell Proliferation Analysis

The CCK‐8 assay (DOJINDO, CK04) and EdU assay (BeyoClick, C0078L) was used to assess the proliferation capacity of Ishikawa and ihESC. In the CCK‐8 assay, cells were serum‐starved for 24 h, followed by the addition of 400 ng mL^−1^ IHH (Abclonal, RP01672) for another 24 h. Subsequently, a 10% CCK‐8 solution was added to each well and incubated at 37 °C for 4 h, following the measurement of absorbance at 450 nm. In the EdU assay, cells were also serum‐starved for 24 h and then treated with 400 ng mL^−1^ IHH for 24 h. After that, 10 µm EdU was added and incubated for 2 h, following the manufacturer's instructions for subsequent fixation, washing, permeabilization, EdU detection, nuclear staining, and fluorescence detection.

### Cell Migration Analysis

A wound healing test was performed to assess the migration of HUVECs. HUVECs were suspended at a concentration of 4 × 10^5^ cells mL and applied to a Culture‐Insert 2 Well (ibidi, 80 209) at a volume of 70 µL. After cell attachment for 24 h, the Culture‐Insert 2 Well was gently removed, and 400 ng mL^−1^ IHH was applied to the cells. Image acquisition was conducted at 0, 6, 12, 24, and 48 h after treatment of IHH, and the scratch area was measured using ImageJ.

### Masson Staining

The paraffin sections were deparaffinized sequentially by environmentally friendly dewaxing transparent liquid (Servicebio, G1128) and ethanol. Masson staining was conducted following the instructions of the Masson tricolor stain set (Servicebio, G1006). The slices were rinsed with 1% acetic acid and rapidly dehydrated with anhydrous ethanol three times. Subsequently, the slides were soaked in xylene for 5 min and finally sealed with neutral resin. Images were taken by NanoZoomer S360 (Hamamatsu, C13220‐01).

### Immunohistochemistry

The paraffin sections were deparaffinized sequentially by environmentally friendly dewaxing transparent liquid (Servicebio, G1128) and ethanol. Then the sections were subjected to antigen retrieval using a citric acid retrieval solution (pH 6.0, Servicebio, G1202). After inhibition of endogenous peroxidase activity with 3% H_2_O_2_, non‐specific binding sites were blocked by incubation with 3% BSA at room temperature for 30 min. The sections were then incubated overnight at 4 °C in a wet box with the primary antibodies diluted in PBS. Afterward, the sections were incubated with the corresponding secondary antibody at room temperature for 50 min. After rinsing with PBS, sections were stained with freshly prepared DAB solution and subsequently counterstained with hematoxylin (blue color), followed by examination under a regular light microscope. The primary antibodies were used as follows: CD11c (CST, 45 581, dilution 1:200); CD4 (Servicebio, GB13588, dilution 1:300); CD8α (Servicebio, GB12068, dilution 1:1600). Image was taken by NanoZoomer S360 (Hamamatsu, C13220‐01).

### Multiplex Immunohistochemistry (mIHC) Staining

mIHC staining was performed using a multiple fluorescent staining kit (Recordbio, RC0086) based on the tyramide signal amplification technology according to the manufacturer's instructions. The antibodies were used as following: E‐cadherin (Proteintech, 20874‐1‐AP, dilution 1:1000); sFRP‐5 (Servicebio, GB114646, dilution 1:1400); IHH (Abcam, ab39634, dilution 1:100); Ki67 (Abcam, ab16667, dilution 1:100); vWF (Servicebio, GB11020, dilution 1:1000); PDGFR‐α (Abcam, ab203491, dilution 1:1000); α‐SMA (Servicebio, GB111364, dilution 1:100); ERα (Santa, sc‐8002, dilution 1:100); Calponin‐1 (Abcam, ab46794, dilution 1:200); collagen III (Proteintech, 22734‐1‐AP, dilution 1:2000). Image was taken by Digital Scanner Pannoramic SCAN II (3DHISTECH, PSCAN22G1101).

### Statistical Analysis

Data are representative of three or more independent experiments. For RNA sequencing data and bench work data, the Shapiro‐Wilk test was used for normality, and the Bartlett test (RNA sequencing data) and F test (bench work data) were used for homogeneity of variances. For normally distributed samples, results were expressed as mean ± standard deviation (SD); otherwise, results were expressed as median (interquartile range). For normally distributed samples with equal variances, parametric tests (t‐test for two groups, ANOVA for multiple groups) were employed; otherwise, non‐parametric tests (Wilcoxon test for two groups, Kruskal–Wallis rank sum test for multiple groups) were used. One‐way ANOVA was used for data with one factor, and two‐way ANOVA was used for data with two factors. Bonferroni's multiple comparisons test was used for multiple comparisons for data with equal variances, and Dunnett's T3 multiple comparisons test was used for multiple comparisons for data without equal variances. R package ggpubr (version 0.6.0) was used for statistical analysis of RNA sequencing data, and GraphPadPrism9.0. was used for statistical analysis of bench work data. The differences were statistically significant with *p < 0.05, **p < 0.01, ***p < 0.001, and ****p < 0.0001.

## Conflict of Interest

The authors declare no conflict of interest.

## Author Contributions

B.L. and J.Q. contributed equally to this work. Y.S. supervised this work. B.L., J.Q., Y.L., Z.S., J.Z., T.Y., Y.Y., Y.M., X.B., X.D., H.G., and Y.H. collected clinical samples. B.L. and J.Q. analyzed the data. B.L., J.Q., and Y.C. performed the experiments. Y.S., W.W., S.H., Y.W., Q.Z., and X.H. provided technical and material support. B.L. and J.Q. drafted the manuscript. Y.S., Y.L., and Z.W. reviewed the manuscript. All authors discussed the results and commented on the manuscript.

## Supporting information



Supporting Information

Supporting Information

## Data Availability

The data that support the findings of this study are openly available in the Genome Sequence Archive^[^ ^107^ ^]^ in National Genomics Data Center,^[^ ^108^ ^]^ China National Center for Bioinformation/Beijing Institute of Genomics, Chinese Academy of Sciences at https://ngdc.cncb.ac.cn/gsa‐human, reference number HRA007399.
