# Supplementary material for: From Invaginating Site to Deep Lesion: Spatial Transcriptomics Unravels Ectopic Endometrial Penetration Features in Adenomyosis
Source: Adv Sci (Weinh). 2025 Apr 7;12(20):2411752. doi: 10.1002/advs.202411752 (PMC12120721; doi:10.1002/advs.202411752)
Supplement: Supplementary file 1 — Supporting Information [file ADVS-12-2411752-s002.docx]

Supporting Information

From invaginating site to deep lesion: spatial transcriptomics unravels ectopic endometrial penetration features in adenomyosis

Boyu Li, Jia Qi, Yumeng Cao, Yijing Long, Zhe Wei, Wang-Sheng Wang, Shuanggang Hu, Yuan Wang, Qinling Zhu, Xiao Hu, Zhe Sun, Jie Zhu, Taiyang Ye, Yejie Yao, Yiwen Meng, Xuejiao Bian, Xinyi Dong, Hengyu Guan, Yunfei Huang, Yun Sun*

**includes:**

Figure S1-10

Legends for Data S1-29

**Other Supporting Information for this manuscript includes the following:**

Data S1-29

**
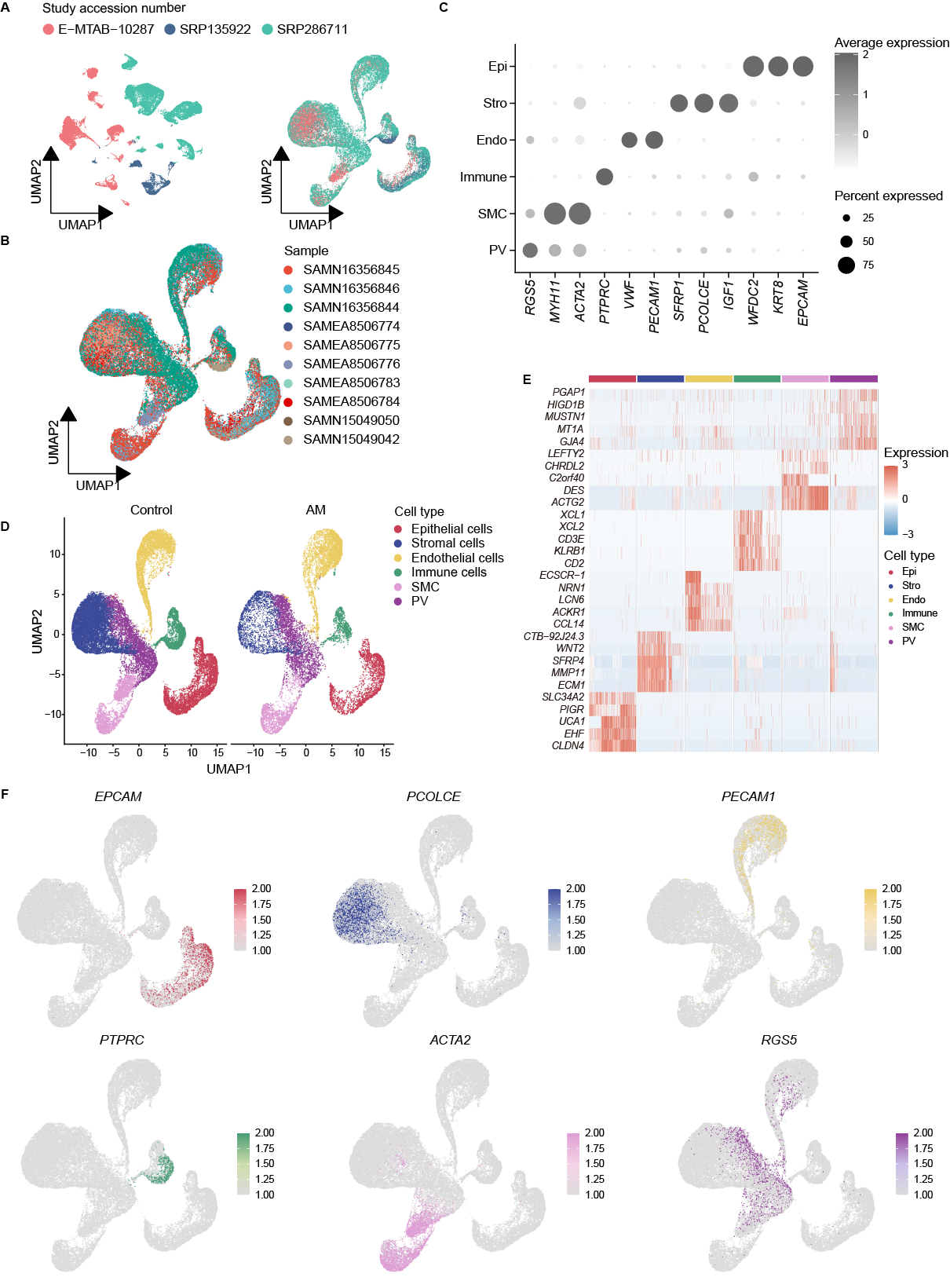
**

**Figure S1.** Integration, clustering, and annotation of single-cell data. A) UMAP plot of single-cell data colored by batches of study before (left) and after (right) batch correction via the CCA. B) UMAP plot of single-cell data colored by samples. C) Dot plot of cell type marker expression level in each cell type. D) UMAP plot colored by cell type and split by group. E) Heatmap showing top genes in each cell type. F) Feature plot showing expression of cell type markers (*EPCAM* for epithelial cells, *PCOLCE* for stromal fibroblasts, *PECAM1* for endothelial cells, *PTPRC* for immune cells, *ACTA2* for SMCs, and *RGS5* for PV).

**
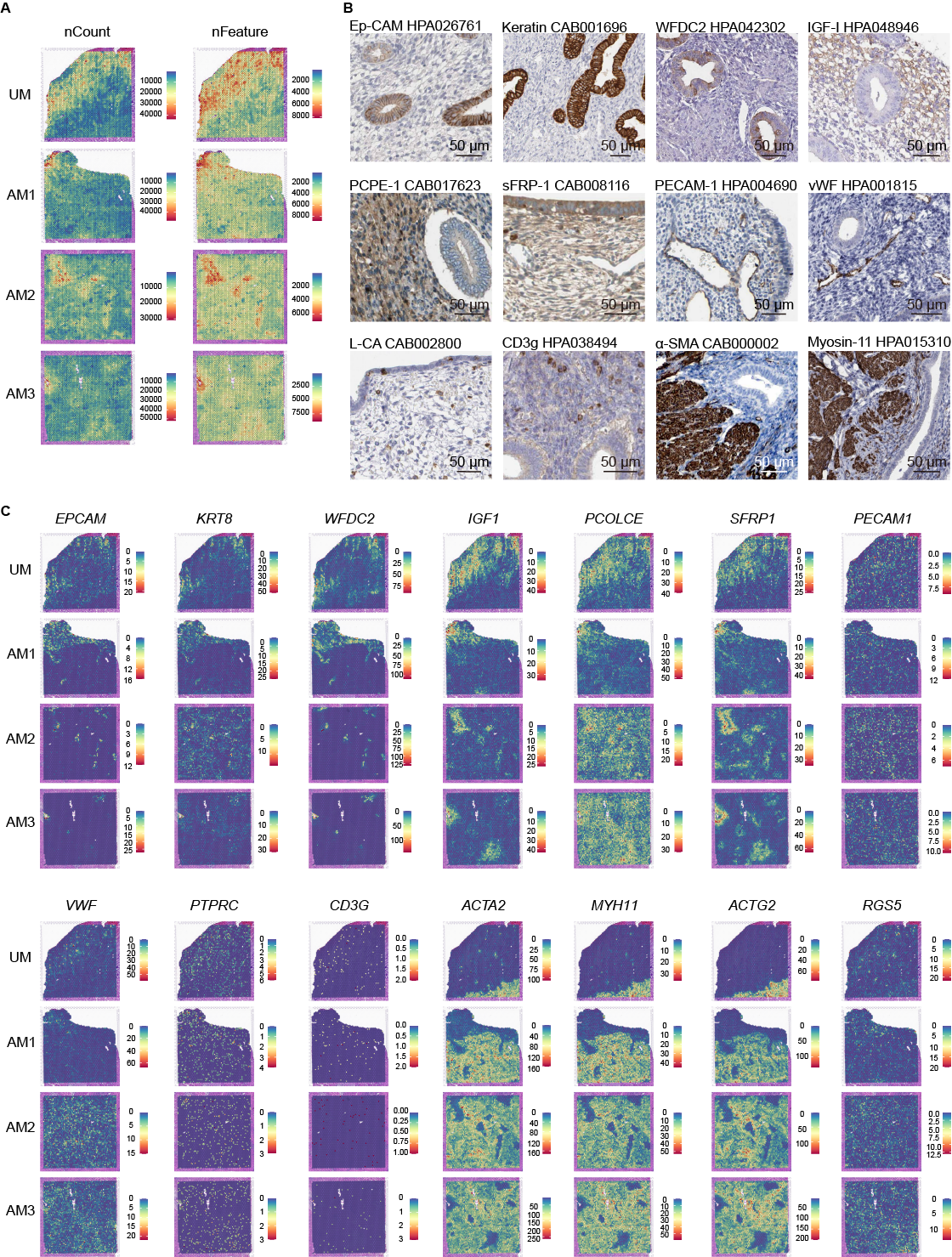
**

**Figure S2.** Spatial expression of cell type markers in Visium data. A) Spatial plot showing the number of counts and features across slices of Visium data. B) Tissue location of cell type markers. The marker of epithelial cells: Ep-CAM, protein of *EPCAM*; Keratin, protein of *KRT8*; WAP four-disulfide core domain protein 2(WFDC2), protein of *WFDC2*. The marker of stromal fibroblasts: IGF-I, protein of *IGF1*; PCPE-1, protein of *PCOLCE*; sFRP-1, protein of *SFRP1*. The marker of endothelial cells: PECAM-1, protein of *PECAM1*; vWF, protein of *VWF*. The marker of immune cells: L-CA, protein of *PTPRC*; CD3g, protein of *CD3G*. The marker of SMCs: a-SMA, protein of *ACTA2*; Myosin-11, protein of *MYH11*. Data was downloaded from The Human Protein Atlas (https://www.proteinatlas.org/). Scale bars: 50 μm. C) Spatial expression of cell type markers across slices.


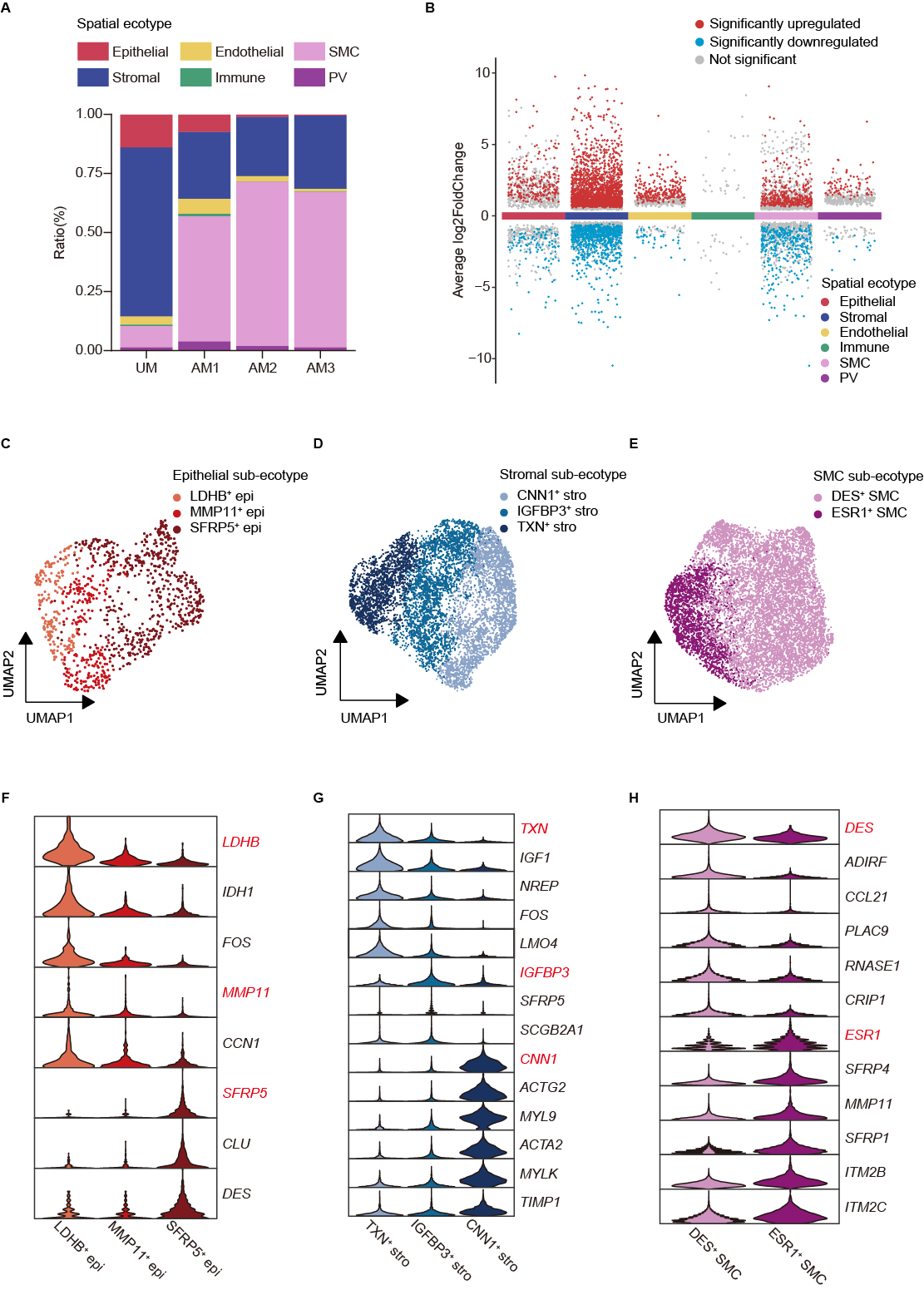


**Figure S3.** Characteristics of spatial ecotypes and re-clustering of epithelial ecotypes, stromal ecotypes, and SMC ecotypes. A) Percentage of these six ecotypes in different slices. B) Volcano plot showing DEGs between group AM and group UM in each spatial ecotype. The statistical analysis was performed by the Wilcoxon test. C-E) UMAP plot of epithelial sub-ecotypes (C), stromal sub-ecotypes (D) and SMC sub-ecotypes (E). F-H) Violin plot showing the expression of marker genes in each epithelial sub-ecotype (F), stromal sub-ecotype (G) and SMC sub-ecotype (H).

**
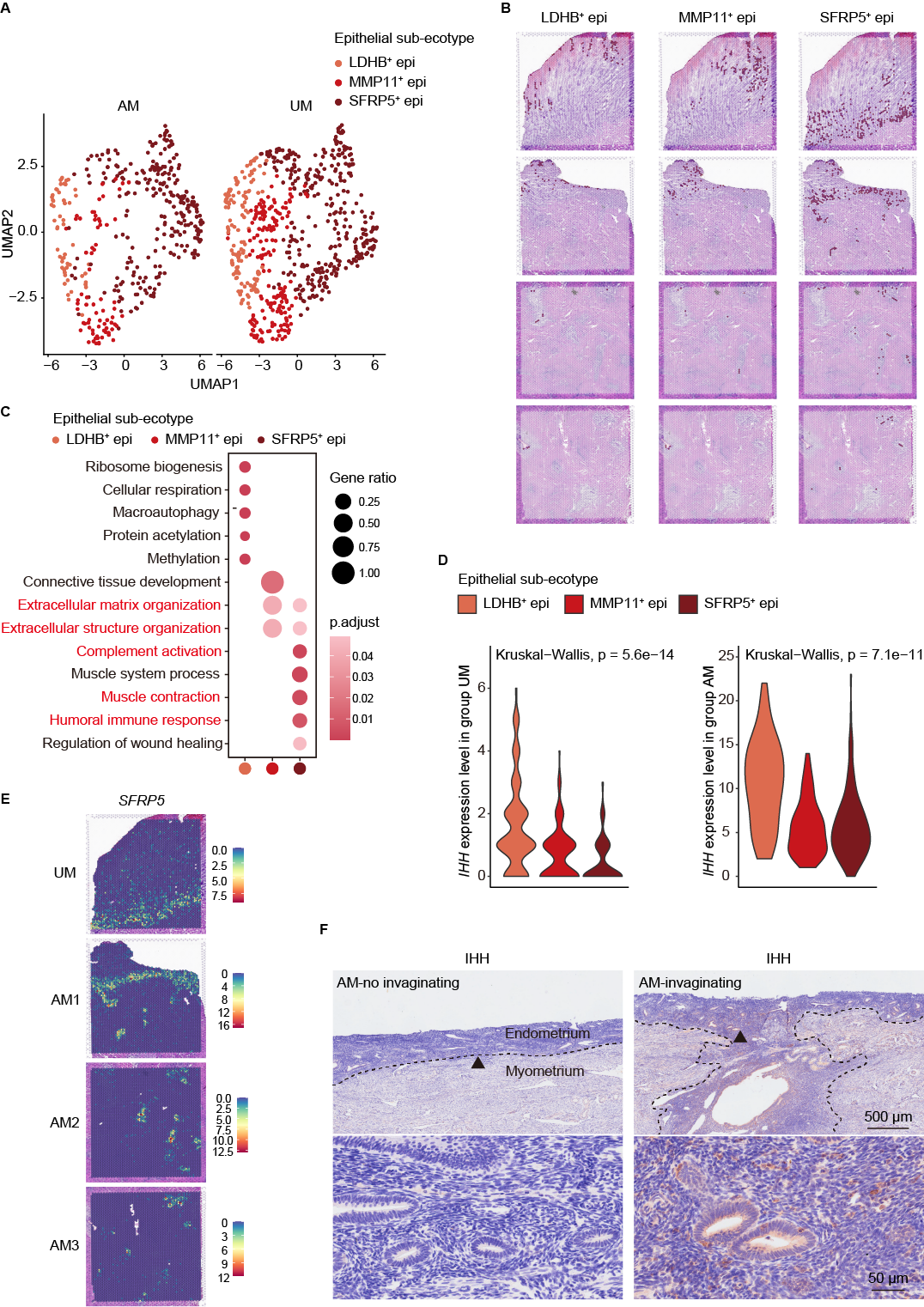
**

**Figure S4.** Characteristic of epithelial ecotypes. A) UMAP plot showing epithelial sub-ecotypes split by group. B) Distribution of LDHB^+^ epithelial ecotypes, MMP11^+^ epithelial ecotypes and SFRP5^+^ epithelial ecotypes, respectively. Indicated epithelial ecotypes are colored in red. C) GOBP terms enriched in each epithelial sub-ecotype. The statistical analysis was performed by Fisher’s test. D) Violin plots showing *IHH* expression level in epithelial sub-ecotypes. Comparisons between epithelial sub-ecotypes in group UM (left) and AM (right) are shown. The Kruskal-Wallis rank sum test was used to obtain p-values. E) Spatial expression of *SFRP5*. SFRP5, a marker of SFRP5^+^ epithelial cells. F) Representative immunohistochemical staining images of adenomyosis samples with (right) or without (left) invaginating structure showing the expression of IHH. The junction of the endometrium and myometrium is delineated with a black dashed line. Black triangles indicate the endometrium-myometrium junction and invaginating site shown at higher magnification. Scale bars: higher magnification, 50 μm; other, 500 μm.


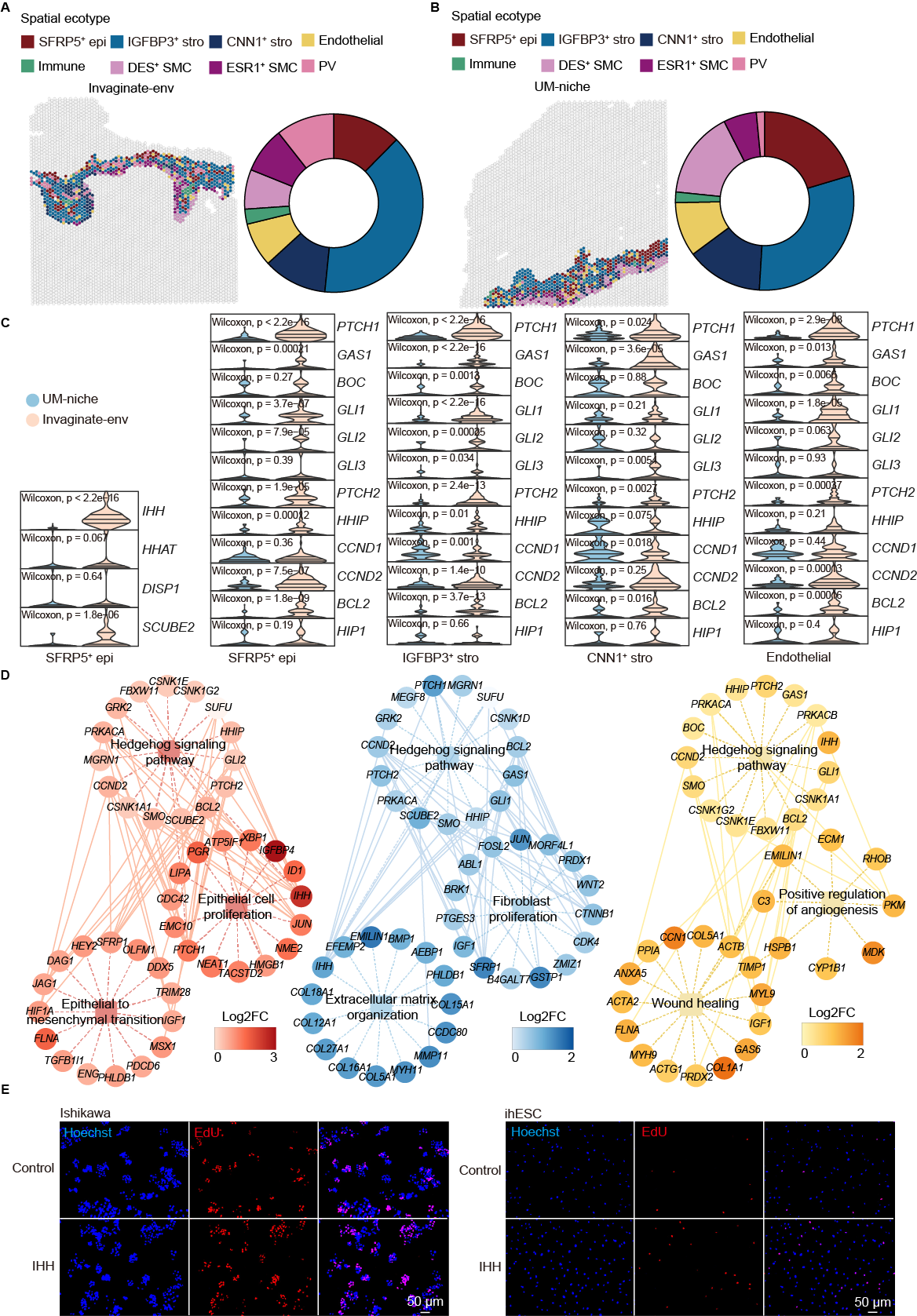


**Figure S5.** Characteristic of the invaginating microenvironment. A-B) Components of invaginating microenvironment (A) and the control niche (B). Distribution (left) and proportion (right) of spatial ecotypes in these two microenvironments are shown. C) Violin plot showing the expression of representative genes related to Hedgehog signaling pathway in indicated ecotypes between invaginating microenvironment and the control niche. Data are presented as the quartiles. P-value was obtained by the Wilcoxon test. D) Molecule interaction networks of genes in the Hedgehog signaling pathway and selected GOBP terms significantly enriched in SFRP5^+^ epithelial ecotypes (red), stromal ecotypes (blue), and endothelial ecotypes (yellow) by STRING. Octagon indicates Hedgehog signaling pathway; round rectangle indicates GOBP terms; ellipse indicates DEGs in invaginating microenvironment compared to the control niche. The statistical analysis for enrichment was performed by Fisher’s test. E) Representative image of EdU assay of Ishikawa and ihESC after treatment with recombinant IHH for 24 h. Scale bars: 50 μm.

**
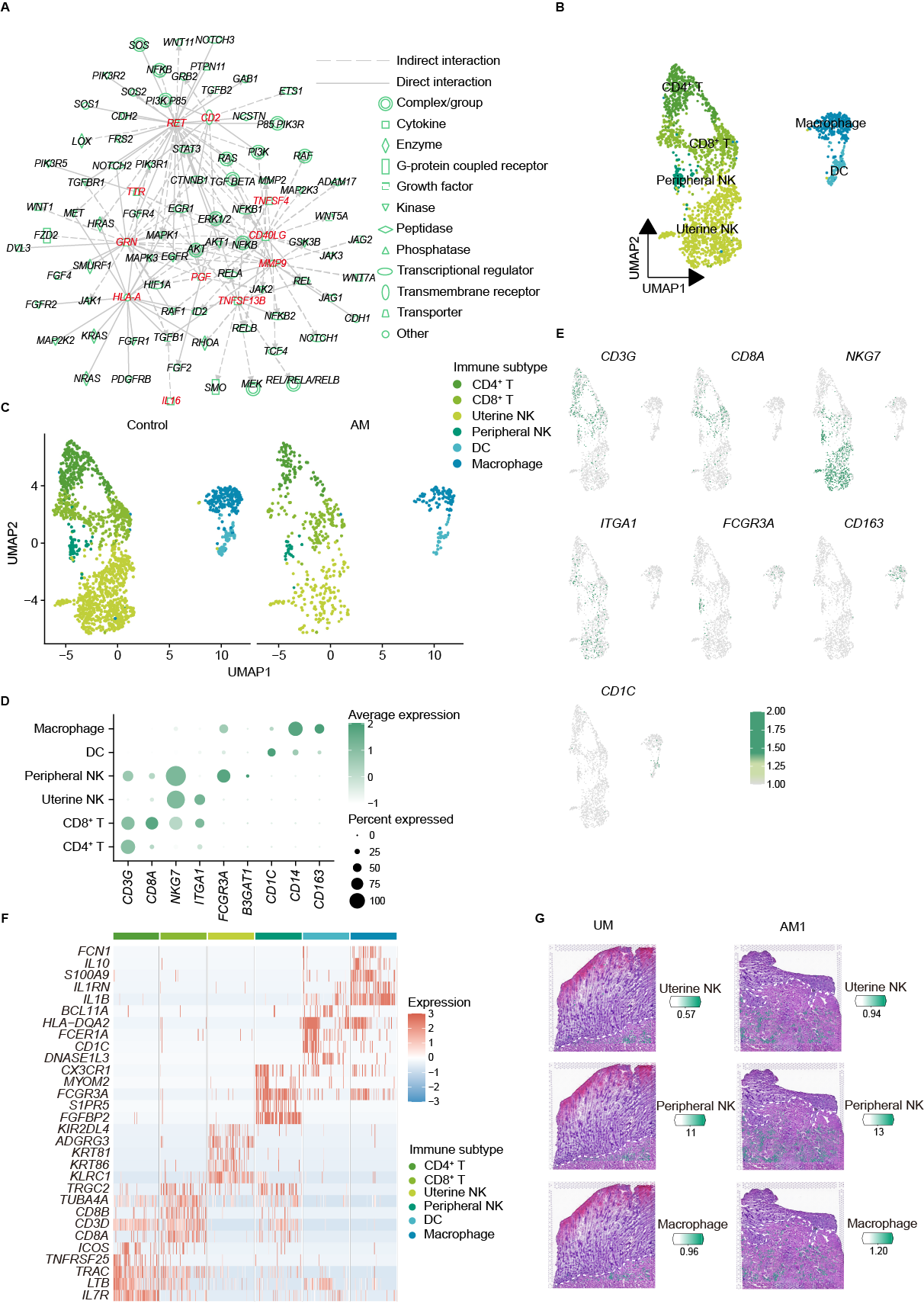
**

**Figure S6.** Re-clustering of immune cells in single-cell data and integration with Visium data. A) Molecular interaction network of immune-derived ligands (colored in red) and EMT-related molecules (colored in black) constructed by IPA. B) UMAP plot of immune subtypes. C) UMAP plot showing immune subtypes split by group. D) Dot plot showing the expression level of cell markers of immune cells (*CD3G* for CD4^+^ T cells; *CD8A* for CD8^+^ T cells; *NKG7* for NK; *ITGA1* for uterine NK; *FCGR3A* and *B3GAT1* for peripheral NK; *CD1C* for DC; *CD14*, *CD163* for macrophage). E) Spatial feature plot of cell type markers. F) Heatmap showing top genes of immune subtypes. G) Cell abundance visualization in spatial coordinates. The junction of the endometrium and myometrium is delineated with a white dashed line.


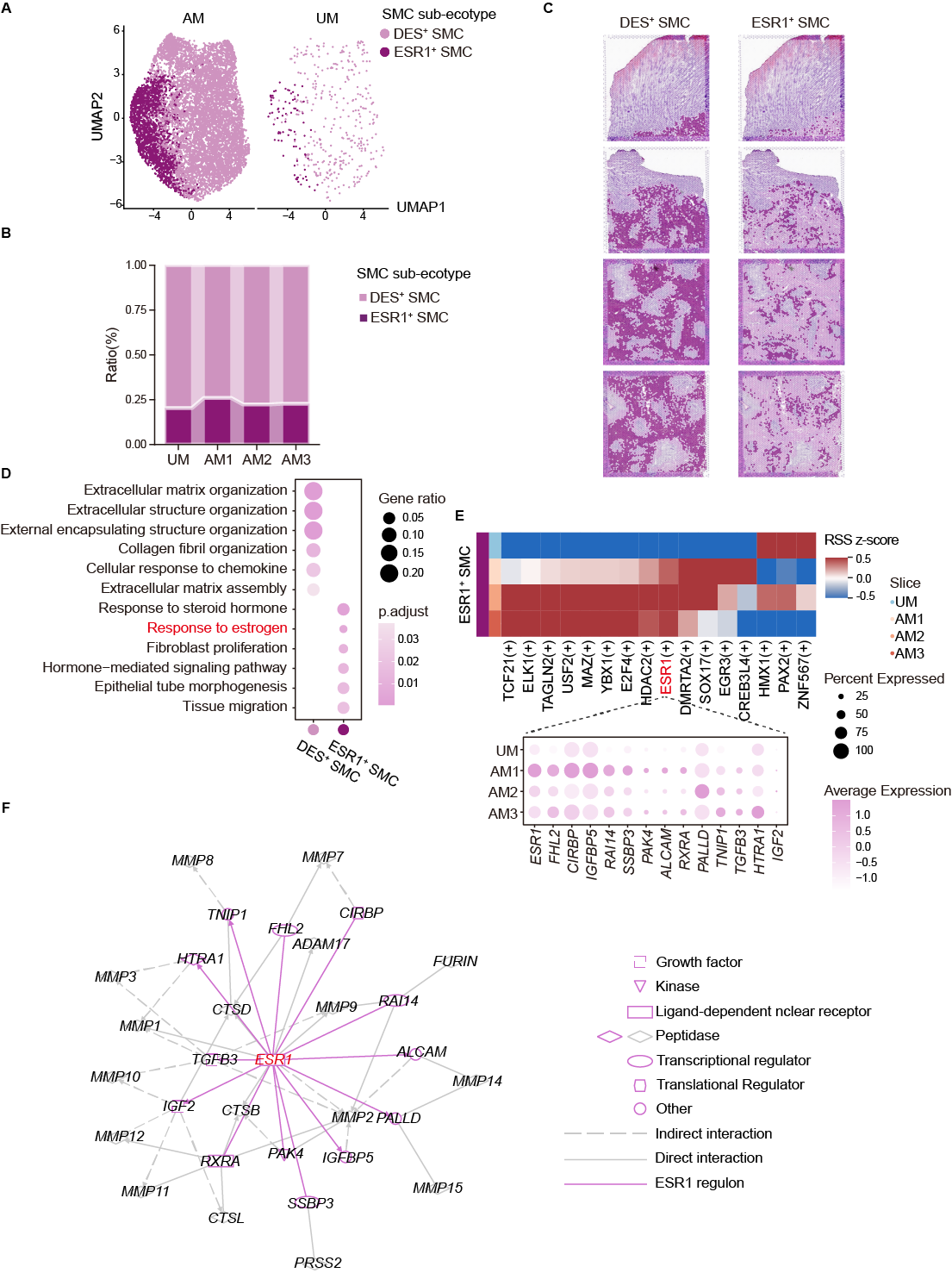


**Figure S7.** Characteristic of SMC ecotypes. A) UMAP plot of SMC sub-ecotypes split by group. B) Percentage of the two SMC sub-ecotypes across slices. C) Distribution of DES^+^ SMC ecotypes and ESR1^+^ SMC ecotypes, respectively. Indicated SMC ecotypes are colored in purple. D) GOBP terms enriched in each SMC sub-ecotype. The statistical analysis was performed by Fisher’s test. E) RSS z-score of specific regulons (upper) and dot plot of genes in ESR1 regulon (bottom) in ESR1^+^ SMC ecotypes across slices according to pySCENIC. F) Molecular interaction network of ESR1 regulon and genes related to collagen degradation constructed by IPA.


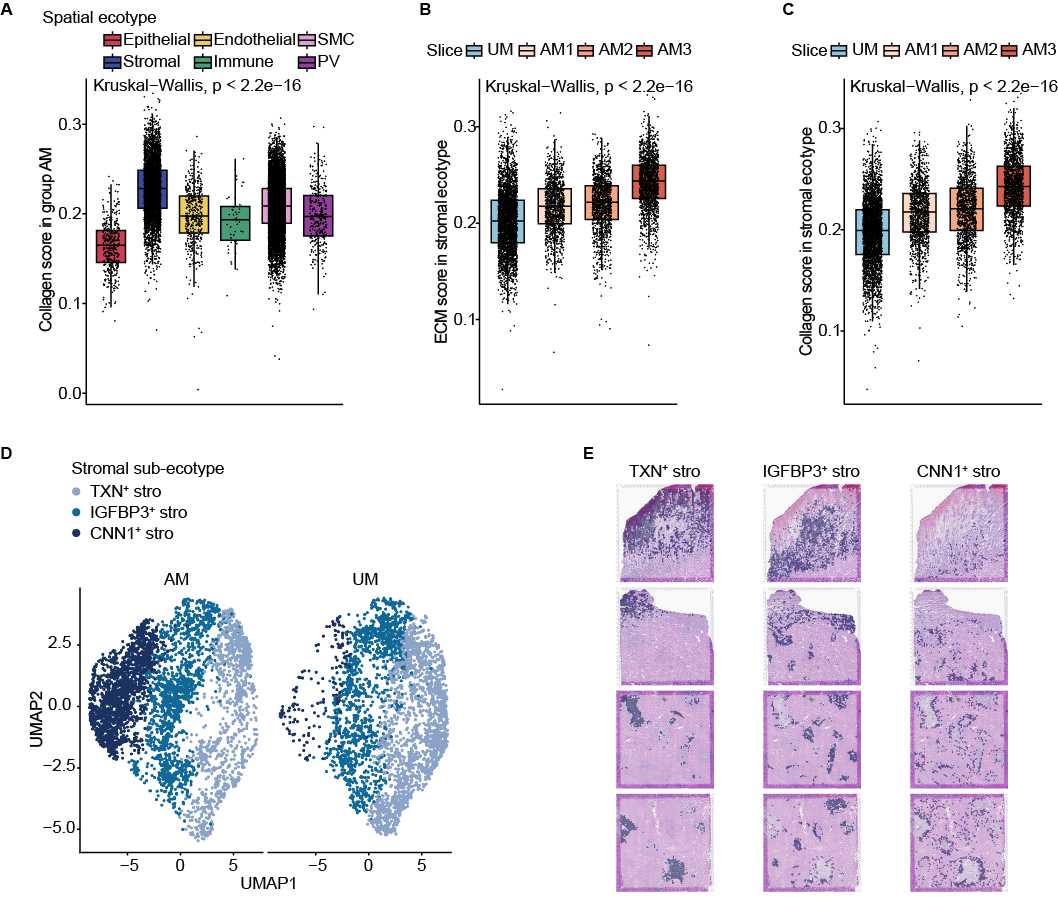


**Figure S8.** Characteristic of stromal ecotypes. A) Box plot showing collagen score between spatial ecotypes in adenomyosis by AUCell. Data are presented as the quartiles. The Kruskal-Wallis rank sum test was used to obtain p-values. B-C) Box plot showing ECM (B) and collagen score (C) in stromal ecotypes across slices. Data are presented as the quartiles. The Kruskal-Wallis rank sum test was used to obtain p-values. D) UMAP plot of stromal sub-ecotypes split by group. E) Distribution of TXN^+^ stromal ecotypes, IGFBP3^+^ stromal ecotypes and CNN1^+^ stromal ecotypes, respectively. Indicated stromal ecotypes are colored in blue.


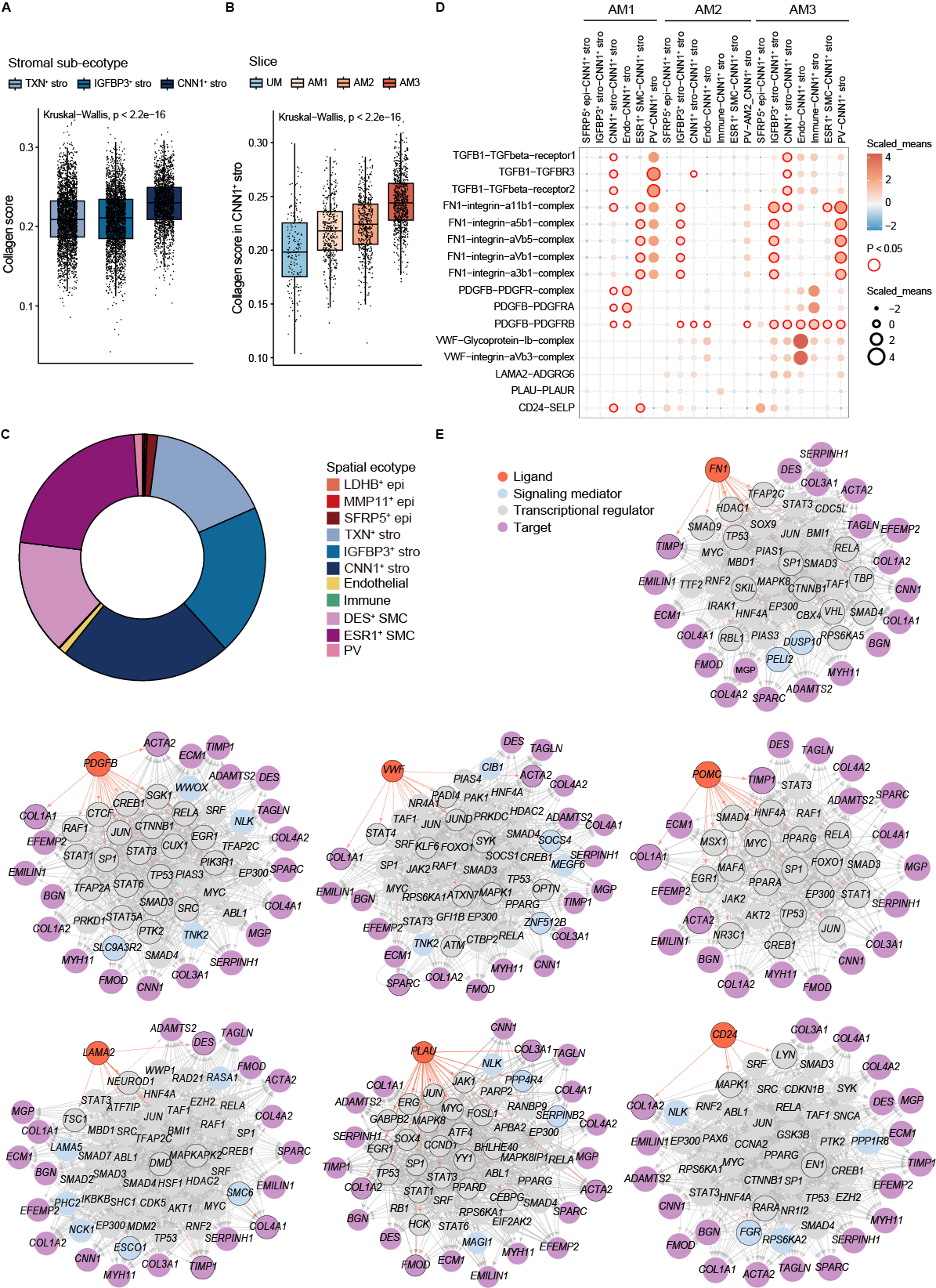


**Figure S9.** Characteristic of CNN1^+^ stromal ecotypes. A) Box plot showing the collagen score of stromal sub-ecotypes. Data are presented as the quartiles. The Kruskal-Wallis rank sum test was used to obtain p-values. B) Box plot showing collagen score of CNN1^+^ stromal ecotypes across slices. Data are presented as the quartiles. The Kruskal-Wallis rank sum test was used to obtain p-values. C) The proportion of spatial ecotypes in lesion microenvironment. D) Fibrosis-related ligand-receptor pairs visualization across AM slices by CellphoneDB. E) The signaling pathways from indicated ligands to fibrosis-related target genes in CNN1^+^ stromal ecotypes inferred by NicheNet. Molecules directly interacted to these ligands are black-circled and indicated by orange arrows.


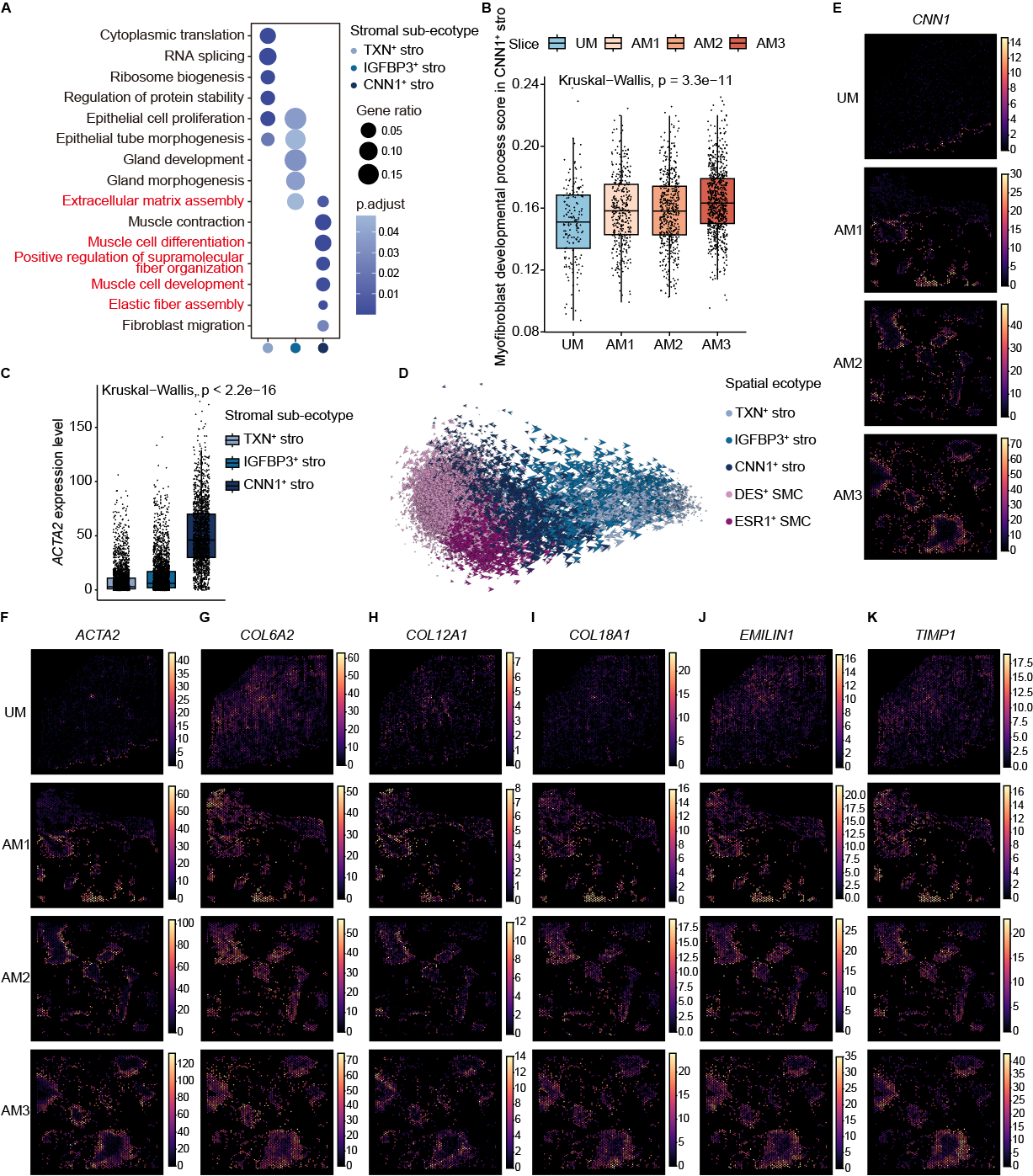


**Figure S10.** FMT in CNN1^+^ stromal ecotypes. A) GOBP terms enriched in each stromal sub-ecotype. The statistical analysis was performed by Fisher’s test**.** B) Box plot showing the expression of gene sets related to myofibroblast developmental process in CNN1^+^ stromal ecotypes across slices by AUCell. Data are presented as the quartiles. The Kruskal-Wallis rank sum test was used to obtain p-values. C) Box plot showing the expression of *ACTA2* in stromal sub-ecotypes. Data are presented as the quartiles. The Kruskal-Wallis rank sum test was used to obtain p-values. D) RNA velocity of stromal and SMC sub-ecotypes on a UMAP embedding according to scVelo. Each arrow shows the direction and speed of movement of an individual spot. E) Spatial expression of *CNN1* (a marker of CNN1^+^ stromal ecotypes) in stromal ecotypes across slices. F) Spatial expression of *ACTA2* (the myofibroblast-associated genes) in stromal ecotypes across slices. G-K) Spatial expression of representative genes of fibrosis in stromal ecotypes across slices.

**Legends**

Data S1. Patient cohort and sample characteristics.

Data S2. Information of single-cell data.

Data S3. Summary of spatially resolved transcriptomics.

Data S4. Cell abundance estimated by cell2location.

Data S5. Spots annotation.

Data S6. DEGs between epithelial sub-ecotypes.

Data S7. GOBP terms enriched in each epithelial sub-ecotype.

Data S8. Canonical pathway activation z-score by ingenuity pathway analysis in epithelial sub-ecotypes (AM vs UM).

Data S9. GOBP terms enriched in each cluster of genes with different pseudo-temporal patterns in epithelial ecotypes.

Data S10-15. DEGs in SFRP5^+^ epithelial ecotypes, IGFBP3^+^ stromal ecotypes, CNN1^+^ stromal ecotypes, endothelial ecotypes, DES^+^ SMC ecotypes, and ESR1^+^ SMC ecotypes (Invaginate_env vs UM_niche), respectively.

Data S16-21. NicheNet ligand prioritized table of SFRP5^+^ epithelial ecotypes, IGFBP3^+^ stromal ecotypes, CNN1^+^ stromal ecotypes, endothelial ecotypes, DES^+^ SMC ecotypes, and ESR1^+^ SMC ecotypes, respectively.

Data S22. GOBP terms enriched in each SMC sub-ecotype.

Data S23. DEGs between ESR1^+^ SMC ecotypes and DES^+^ SMC ecotypes.

Data S24. GOBP terms enriched in each gene cluster of ESR1^+^SMC ecotypes in different groups.

Data S25. GOBP terms enriched in each cluster of genes with different pseudo-temporal patterns in stromal ecotypes.

Data S26. DEGs between stromal sub-ecotypes.

Data S27. GOBP terms enriched in each stromal sub-ecotype.

Data S28. RSS z-score of regulons in stromal sub-ecotypes.

Data S29. RSS z-score of regulons in CNN1^+^ stromal ecotypes across slices.
